# Silencing of *StRIK* in potato suggests a role in periderm related to RNA processing and stress

**DOI:** 10.1186/s12870-021-03141-z

**Published:** 2021-09-07

**Authors:** Pau Boher, Marçal Soler, Sandra Fernández-Piñán, Xènia Torrent, Sebastian Y. Müller, Krystyna A. Kelly, Olga Serra, Mercè Figueras

**Affiliations:** 1 Laboratori del Suro, Biology Department, Universitat de Girona, Campus Montilivi, E-17071 Girona, Catalonia Spain; 2 Department of Plant Sciences, University of Cambridge, Downing Street, Cambridge, CB2 3EA UK

**Keywords:** RS2-INTERACTING KH PROTEIN, Potato tuber periderm, Flowering, RNA regulation, KH-domain RNA-binding protein

## Abstract

**Background:**

The periderm is a protective barrier crucial for land plant survival, but little is known about genetic factors involved in its development and regulation. Using a transcriptomic approach in the cork oak (*Q. suber*) periderm, we previously identified an *RS2-INTERACTING KH PROTEIN* (*RIK*) homologue of unknown function containing a K homology (KH)-domain RNA-binding protein, as a regulatory candidate gene in the periderm.

**Results:**

To gain insight into the function of RIK in the periderm, potato (*S. tuberosum*) tuber periderm was used as a model: the full-length coding sequence of *RIK*, hereafter referred to as *StRIK*, was isolated, the transcript profile analyzed and gene silencing in potato performed to analyze the silencing effects on periderm anatomy and transcriptome. The *StRIK* transcript accumulated in all vegetative tissues studied, including periderm and other suberized tissues such as root and also in wounded tissues. Downregulation of *StRIK* in potato by RNA interference (*StRIK-*RNAi) did not show any obvious effects on tuber periderm anatomy but, unlike Wild type, transgenic plants flowered. Global transcript profiling of the *StRIK*-RNAi periderm did show altered expression of genes associated with RNA metabolism, stress and signaling, mirroring the biological processes found enriched within the in silico co-expression network of the Arabidopsis orthologue.

**Conclusions:**

The ubiquitous expression of *StRIK* transcript, the flower associated phenotype and the differential expression of *StRIK*-RNAi periderm point out to a general regulatory role of StRIK in diverse plant developmental processes. The transcriptome analysis suggests that StRIK might play roles in RNA maturation and stress response in the periderm.

**Supplementary Information:**

The online version contains supplementary material available at 10.1186/s12870-021-03141-z.

## Background

Plants cope with ever-changing environmental factors which are sometimes adverse and hinder their survival. To deal with these unfavorable circumstances, plants rely on developmental solutions and regulatory networks to protect their body structure and to optimize their metabolism and physiology. Among the adaptations that land plants develop, the formation of waterproof barriers is essential to prevent uncontrolled water loss [1] and pathogen attack [2]. This protection is achieved in secondary organs, tubers and wounded tissues by an external barrier known as the periderm. The periderm consists of three different layers from inside to outside: the parenchymatous phelloderm, the meristematic phellogen and the cork or phellem. The phellem confers protection to the periderm through depositing suberin, lignin and associated-waxes within the cell walls [3]. Despite the importance of periderm ontogenesis for land plant survival, the molecular networks that regulate its formation and differentiation are little known. Several transcriptomic studies have used cork tissue [4–12] resulting in a substantial list of candidate regulatory proteins including phytohormone-related proteins, signal transductors and transcriptional regulators. Several transcription factors were shown to be relevant in periderm for suberin deposition: *StNAC103*, *QsMYB1* and *ANAC046* [13–16]. *StNAC103* was suggested as a repressor of suberin and associated waxes in potato tuber periderm [15, 16], while *QsMYB1* and *ANAC046* were proposed as inducers of suberin deposition in cork oak (*Q. suber*) bark and Arabidopsis root periderm, respectively [13, 14].

In a previous transcriptomic study in cork oak, a gene homologous to the maize *RS2-INTERACTING KH PROTEIN* (*RIK*) [17] was upregulated in cork compared to xylem tissue [7]. The RIK protein interacted with the maize gene *rough sheath2* (*rs2*), the orthologue of Arabidopsis *ASYMMETRIC LEAVES 1* (*AS1*) which forms conserved complexes with *ASYMMETRIC LEAVES 2* (*AS2*) and the histone chaperone *HIRA* [17]*.* The AS1/AS2/HIRA complex maintains the silencing of class I KNOX genes through a repressed chromatin state, promoting stem cell activity and meristem maintenance to form determinate lateral organs [17–20]. It was hypothesized that RIK could contribute to the epigenetic repression of *KNOX* genes in the AS1/AS2/HIRA complex by binding of regulatory RNAs [17], although the function of the RIK protein remains to be experimentally determined. Sequence analysis of RIK revealed that it contains a K-homology (KH) RNA binding domain and a like helicase domain (LHD) [17]. KH domain-containing proteins are RNA binding proteins known to be involved in splicing, regulation of post-transcriptional gene expression, mRNA stability, miRNA biogenesis and heterochromatin silencing [21, 22]. The RIK protein is encoded by a single gene in Arabidopsis, maize and rice and has a Splicing Factor 1-like KH domain, although the canonical core sequence of KH domain is weakly conserved among the RIK proteins [23]. The phylogenetic tree shows that RIK proteins form a distinct clade, distinguished from all other SF1-like KH and KH proteins. Although the *RIK* transcript accumulates in all tissues analyzed in maize, a higher level of mRNA accumulation was shown in the shoot apical meristem and a lower level in older leaves [23]. In plants, several studies have demonstrated that KH domain proteins influence flower development [24–26], vegetative growth [27], stress tolerance [28] and jasmonate signaling [29].

Here, to get closer to the function of the potato *StRIK* gene in periderm, *StRIK* was stably silenced and the tuber periderm anatomy and transcriptome were analyzed. *StRIK* downregulation affected the expression of genes related to RNA metabolism, stress response and signaling in tuber periderm.

## Results

### StRIK and its orthologues show two SF1_like-KH domains

The StRIK protein sequence, translated from the cDNA sequence isolated from *S. tuberosum* Group Tuberosum cv Désirée, shows significant homology (98.82, 97.85, 97.85, 98.13% identities) with the four protein isoforms encoded in PGSC0003DMG400025145 locus (corresponding to PGSC0003DMP400043638, PGSC0003DMP400043639, PGSC0003DMP400043637, PGSC0003DMP400043640, respectively) from the *S. tuberosum* Group Phureja genome [30]. The Conserved Domain Database [31] identified two SF1-like KH (cd02395) domains located between the amino acids 127–203 (e-value 1.38 e^− 04^) and 216–291 (e-value 1.77 e^− 04^) (Fig. S1). As shown in Fig. S1, the KH domain core sequence is highly conserved amongst all the RIK proteins included in the alignment (V/IRGPNDQYI) but, as it occurs in the maize RIK protein [23], it is weakly conserved with the canonical IIGxxGxxI core sequence of the KH domains [21].

### *StRIK* transcript is ubiquitous

The transcript profile of *StRIK* in potato tissues analyzed by RT-qPCR showed high transcript levels in root, stem, leaf, tuber flesh and tuber periderm (Fig. S2A). These results confirmed the ubiquitous expression of the gene found in the RNA-seq data available from the *S. tuberosum* Group Phureja [30], which also revealed moderate gene induction in flower organs (flower, petiole and stamen), in root and in tissues with meristematic activity (shoot apex, tuber sprout) (Fig. S2B). Moreover, *S. phureja RIK* is induced by abiotic stresses such as mannitol (osmotic stress), water-stressed leaves, salt and abscisic acid (ABA) treatments while it is downregulated upon heat and cytokinin (BAP; benzyl adenine) treatments (Fig. S2B). The effect of wounding (see Plant Material subsection) on transcript abundance of *StRIK* was analyzed. According to the regression analysis there was a highly significant linear increase in *StRIK* levels after wounding (*p* < 0.001) (Fig. S2C).

### StRIK is located in the nucleus

**Fig. 1:**
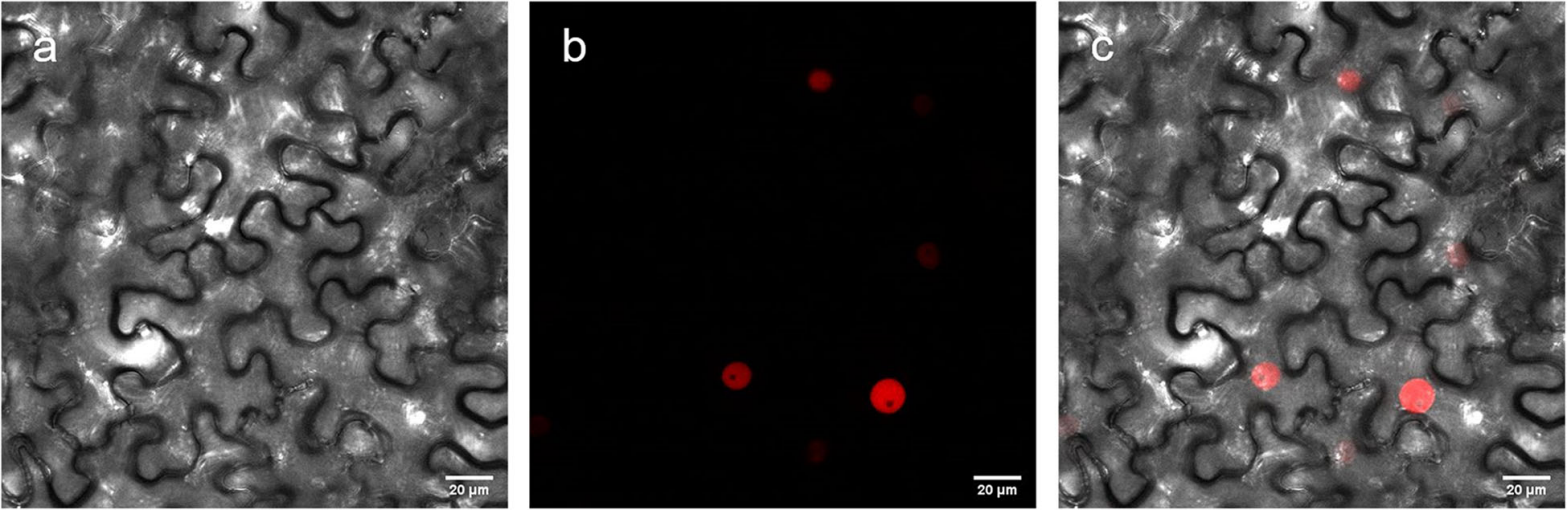
Subcellular localization of RFP-StRIK in *N. benthamiana* leaf. Micrographs obtained of **a**) bright field, **b**) red fluorescence channel and **c**) overlay of bright field and red fluorescence channel

The subcellular localization of StRIK protein was determined in *N. benthamiana* by transient *Agrobacterium*-mediated leaf transformation to yield StRIK tagged with RFP to the N-terminal end. After 72 h of infection, a red fluorescence, indicative of the StRIK protein accumulation, was detected concentrated in a single spot showing the typical pattern of nuclear located proteins with the gap free of labeling corresponding to the nucleolus (Fig. 1) [32].

### *StRIK* silencing does not affect phellem anatomy but induces flowering

To evaluate the contribution of *StRIK* to phellem formation, *StRIK* was silenced using RNAi (Fig. S3). A 246 bp fragment spanning the nucleotides 758 to 1003 of the *StRIK* coding sequence (Genbank accession number: MT622318) was used, which overlaps partially or completely with exons 8, 9 and 10 of the gene (Fig. S4). To check the possibility of off-target silencing we performed a BLASTN analysis using the silencing RNAi sequence as query against the Potato Genome Database [30] setting the expected threshold parameter to 1. The analysis identified the representative transcript (PGSC0003DMT400064730) and two transcript isoforms of *RIK* as RNAi-targets (PGSC0003DMT400064729 and PGSC0003DMT400064731), while the fourth and shortest predicted *RIK* transcript isoform (PGSC0003DMT400064735) was not identified because is not targeted by the RNAi fragment (Fig. S4 and Table S1). The other transcripts identified by the BLASTN analysis showed a partial match in 18 or less consecutive nucleotides, hence cross-silencing was unlikely.

**Fig. 2:**
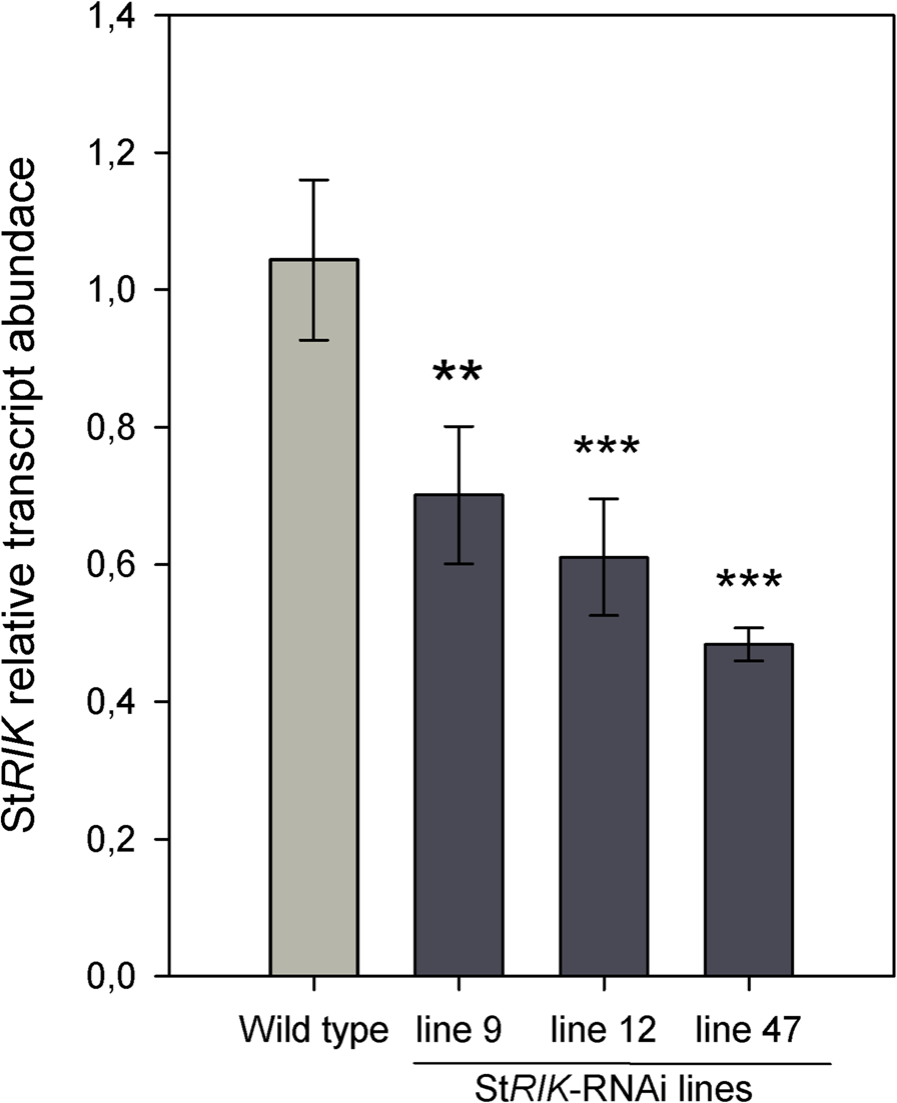
*StRIK* transcript accumulation in the periderm of Wild type and *StRIK-*RNAi lines. Three independent transformation events were analyzed (lines 9, 12 and 47) and for each line, three biological replicates were used. For each biological replicate, we used three technical replicates (Dunnett’s test for comparing multiple groups to a control was used (two asterisks (**, *P* < 0.01), three asterisks (***, *P* < 0.001))

**Fig. 3:**
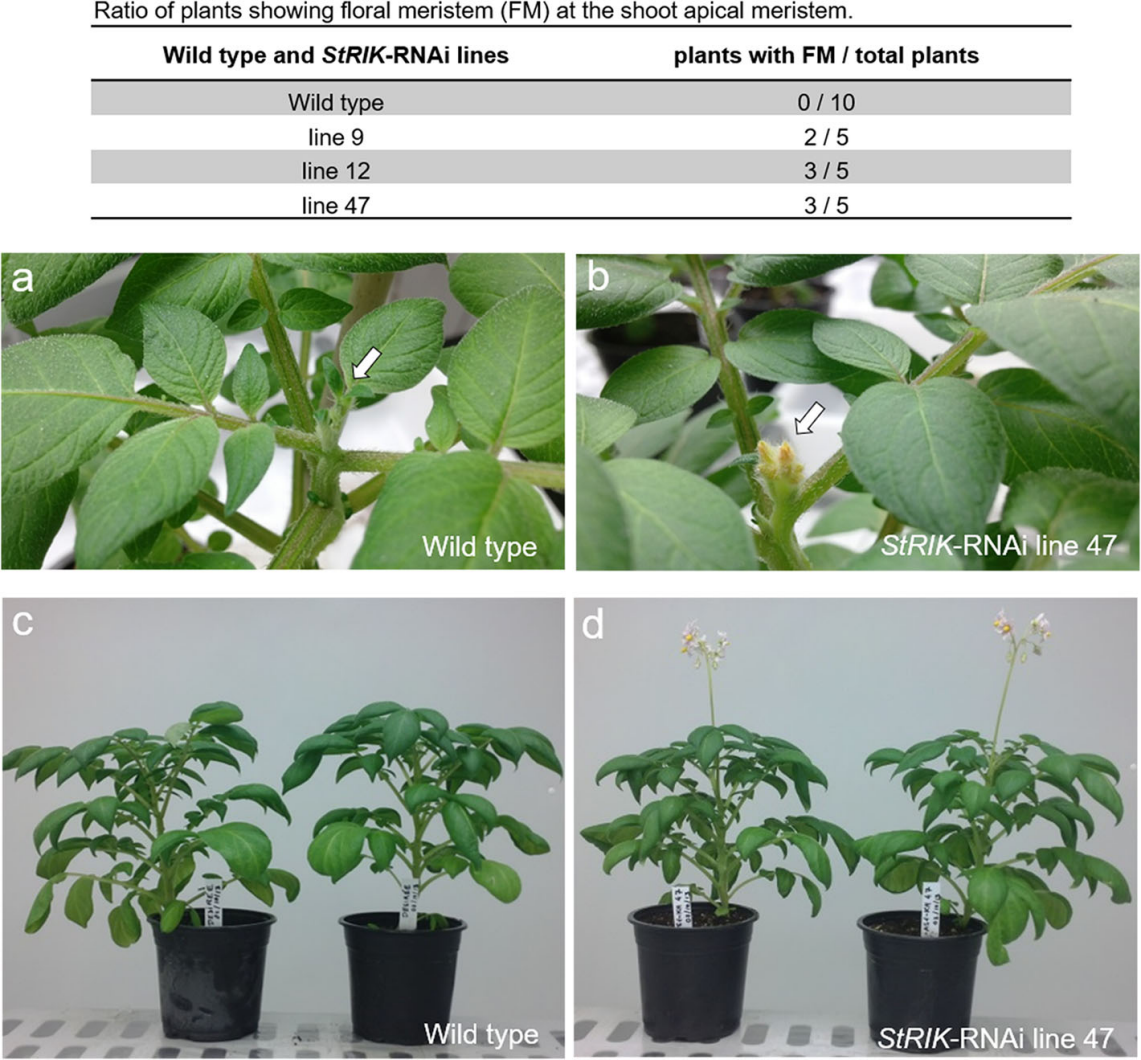
Effects of *StRIK* silencing in flower development. Ratio of plants showing flower meristem (FM) per total plants for Wild type and *StRIK* silenced lines. The arrows point to the detail of **a**) the shoot apical meristem in Wild type plant and **b**) the flower meristem in *StRIK-*RNAi line. Full view of adult potato plants showing differences in their flowering capacity between **c**) the Wild type and **d**) *StRIK*-RNAi lines. The co-authors are the owners of the images

**Fig. 4:**
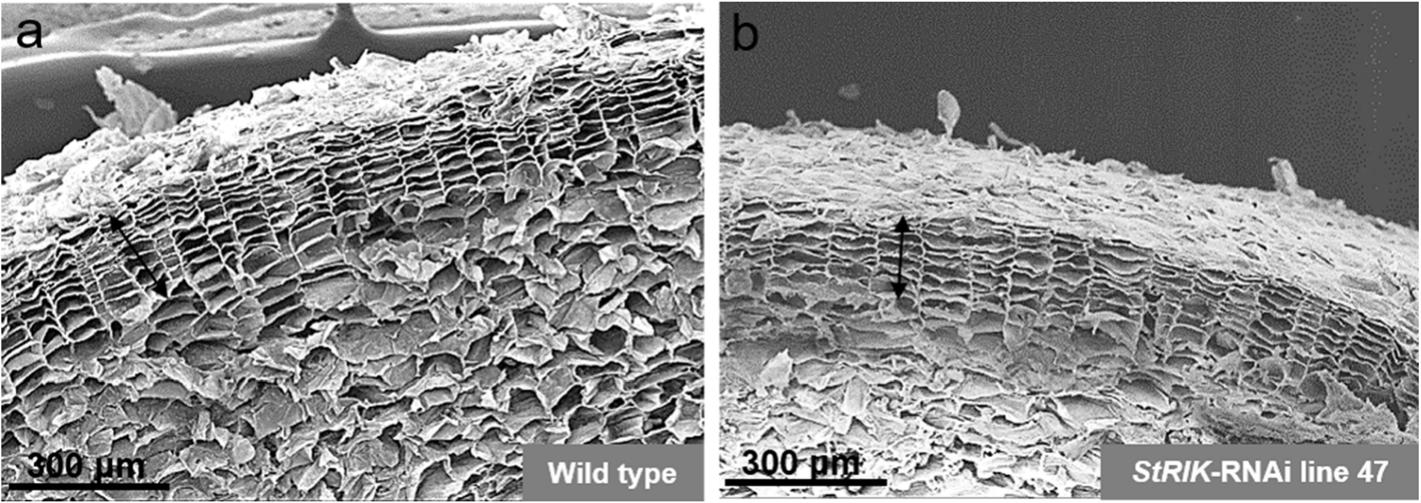
Effects of *StRIK* silencing in periderm anatomy. SEM micrograph of tuber periderm cross-section of **a**) Wild type and **b**) *StRIK*-RNAi lines. Similar number of cell layers and phellem organization was observed in both lines. Phellem is shown with a black arrow

Twenty independent transformation events producing *StRIK*-RNAi kanamycin-resistant potato plants were analyzed by RT-qPCR. Five transgenic lines displayed a reduction in *StRIK* transcript levels in leaves (Fig. S5a) and tuber periderm (Fig. S5b). The *StRIK*-RNAi lines 9, 12, and 47 were propagated to produce enough tubers for subsequent phenotypic and transcriptomic analyses. The RT-qPCR was repeated in these three lines and the silencing of *StRIK* in periderm was confirmed (Fig. 2). When five *StRIK*-RNAi plants from each of the three lines (line 9, 12 and 47) were grown in soil under long-day conditions (12 h light/12 h dark), 53.3% of them flowered whereas, as expected, none of the Wild type (0 out of 10) flowered because the Désirée cultivar does not flower in our growth conditions (Fig. 3a). Evident floral transition at the shoot apical meristem was observed in the *StRIK*-RNAi lines unlike Wild type (Fig. 3b compared with Fig. 3a), and fully developed flowers were formed (Fig. 3d compared with Fig. 3c). The anatomy of the potato periderm was investigated in Wild type and *StRIK* silenced lines of 21-d stored tubers. Scanning Electron Microscopy (SEM) did not reveal obvious differences in the number of cell layers or in the general cellular architecture (Fig. 4).

### The periderm transcriptome comparison shows that *StRIK* silencing affects RNA metabolism, transposon- and stress-related genes

To explore the effects of *StRIK* silencing on the global transcription profile, the periderm RNA from three replicates of each of the three *StRIK*-RNAi lines (lines 9, 12 and 47) and Wild type potato tubers was extracted and sequenced using an Illumina HiSeq2000. Reads were mapped to the potato transcriptome and the number of reads per transcript was quantified. To identify those genes showing differential expression between *StRIK* silenced and Wild type plants, we used the six *StRIK*-RNAi libraries where the *StRIK* abundance was less than two thirds that of the Wild type (line 9 *n* = 1, line 12 *n* = 2; line 47 *n* = 3). A total of 101 differentially expressed genes (DEGs) were identified, 66 genes were upregulated and 35 genes were downregulated in *StRIK-*RNAi lines (Table S2). Using the potato gene identifier, Uniref100 (downloaded on 16/06/2017) (Suzek et al., 2007) and TAIR (Arabidopsis Information Resource (https://www.arabidopsis.org/)), functional annotations were retrieved through the Spud DB Potato Genomics Resources (http://solanaceae.plantbiology.msu.edu/pgsc_download.shtml) [30]. Taking advantage of the information in these Genomic Resources, the DEGs were classified manually into functional categories. DEGs showing log_2_FC values ≤ − 2 and ≥ 2 in the main functional groups identified are shown in Table 1. The biological processes dominated by genes upregulated in *StRIK*-RNAi lines were RNA metabolism, proteolysis and metabolism while stress, transposable elements and signaling were biological processes with similar numbers of up and downregulated genes in the *StRIK*-RNAi lines (Table S2).

**Table 1:** DEGs between Wild type (WT) and *StRIK*-RNAi (RIK) tuber periderm

Functional group	Normalized reads		DE	Uniref gene Description	Best BLASTP TAIR
	WT (1)	*StRIK*-RNAi (2)			
*RNA metabolism*					
PGSC0003DMT400063700	47	747	2 > 1	RNase H family protein	AT5G35695
PGSC0003DMT400071451	63	729	2 > 1	RRNA intron-encoded homing endonuclease	-
PGSC0003DMT400091537	280	2636	2 > 1	RRNA intron-encoded homing endonuclease	-
PGSC0003DMT400085242	214	2014	2 > 1	RRNA intron-encoded homing endonuclease	-
PGSC0003DMT400079124	0	3	2 > 1	RNA recognition motif-containing protein	AT5G19030
PGSC0003DMT400063698	0	15	2 > 1	RNase H family protein	-
PGSC0003DMT400094705	509	6089	2 > 1	RRNA intron-encoded homing endonuclease	-
*Transposable elements (TE)-related genes*					
PGSC0003DMT400046437	20	1	1 > 2	TNP2, partial	-
PGSC0003DMT400041204	34	485	2 > 1	Retrotransposon protein	AT5G41980
PGSC0003DMT400092517	47	1	1 > 2	LINE-type retrotransposon LIb DNA, complete sequence, Insertion at the S14 site	-
PGSC0003DMT400085583	0	5	2 > 1	Transposon MuDR mudrA	-
PGSC0003DMT400039369	1188	296	1 > 2	Transposase	-
PGSC0003DMT400003803	6	92	2 > 1	Transposase	AT1G43722
*Stress*					
PGSC0003DMT400083063	1	48	2 > 1	Ribulose bisphosphate carboxylase large chain	ATCG00490
PGSC0003DMT400026705	3	30	2 > 1	11S globulin isoform 4	AT5G44120
PGSC0003DMT400080875	2528	27100	2 > 1	Senescence-associated protein	-
PGSC0003DMT400013091	0	4	2 > 1	PR-1	AT3G19690
PGSC0003DMT400005448	0	5	2 > 1	Vacuolar H+-pyrophosphatase	AT1G15690
PGSC0003DMT400024872	0	12	2 > 1	K+ channel inward rectifying	AT5G46240
PGSC0003DMT400061541	3	60	2 > 1	Dehydration responsive element binding protein	AT5G05410
*Proteolysis*					
PGSC0003DMT400026261	316	3520	2 > 1	Nb cell deth marker	AT1G17860
PGSC0003DMT400026262	439	6499	2 > 1	Conserved gene of unknown function	AT1G17860
PGSC0003DMT400024552	3	38	2 > 1	Gene of unknown function	AT1G50690
PGSC0003DMT400049320	0	4	2 > 1	Ulp1 protease family, C-terminal catalytic domain containing protein	-
PGSC0003DMT400040325	16	2	1 > 2	F-box family protein	AT3G07870
*Signaling*					
PGSC0003DMT400047864	5	37	2 > 1	Signal transducer	AT1G67900
PGSC0003DMT400001990	2	8	2 >1	Calcium/calmodulin-dependent protein kinase CaMK3	AT3G50530
PGSC0003DMT400047866	16	79	2 > 1	Signal transducer	AT1G67900
PGSC0003DMT400054991	0	4	2 > 1	Calcium-dependent protein kinase 20	AT2G38910
PGSC0003DMT400047867	14	71	2 > 1	Signal transducer	AT1G67900
*Metabolism*					
PGSC0003DMT400052839	1	18	2 > 1	Beta-amylase PCT-BMYI	AT4G17090
PGSC0003DMT400049003	686	12004	2 > 1	Cytochrome P450 like_TBP	-
PGSC0003DMT400070122	5	26	2 > 1	Glucosyltransferase	AT4G36770
PGSC0003DMT400014902	3	0	1 > 2	Cytochrome P450	AT1G12740
PGSC0003DMT400019561	0	5	2 > 1	Cytochrome P450	AT5G36110
*Acyl lipid Metabolism*					
PGSC0003DMT400042102	1	10	2 > 1	Acetyl-coenzyme A carboxylase carboxyl transferase alpha	AT2G38040
PGSC0003DMT400079076	0	3	2 > 1	Acyl-[acyl-carrier-protein] desaturase	AT1G43800

List of genes showing log_2_FC values ≤ -2 and ≥ 2 classified in the main functional groups. For each gene the mean of normalized reads (effective counts) found in Wild type replicates (*n* = 3) and *StRIK*-RNAi (line 9 *n* = 1; line 12 *n* = 2; line 47 *n* = 3) is shown. Also, it is reported the potato gene identifier, the Uniref gene identifier and the best BLASTP in TAIR (e value ≤ 10^-5^)

**Fig. 5:**
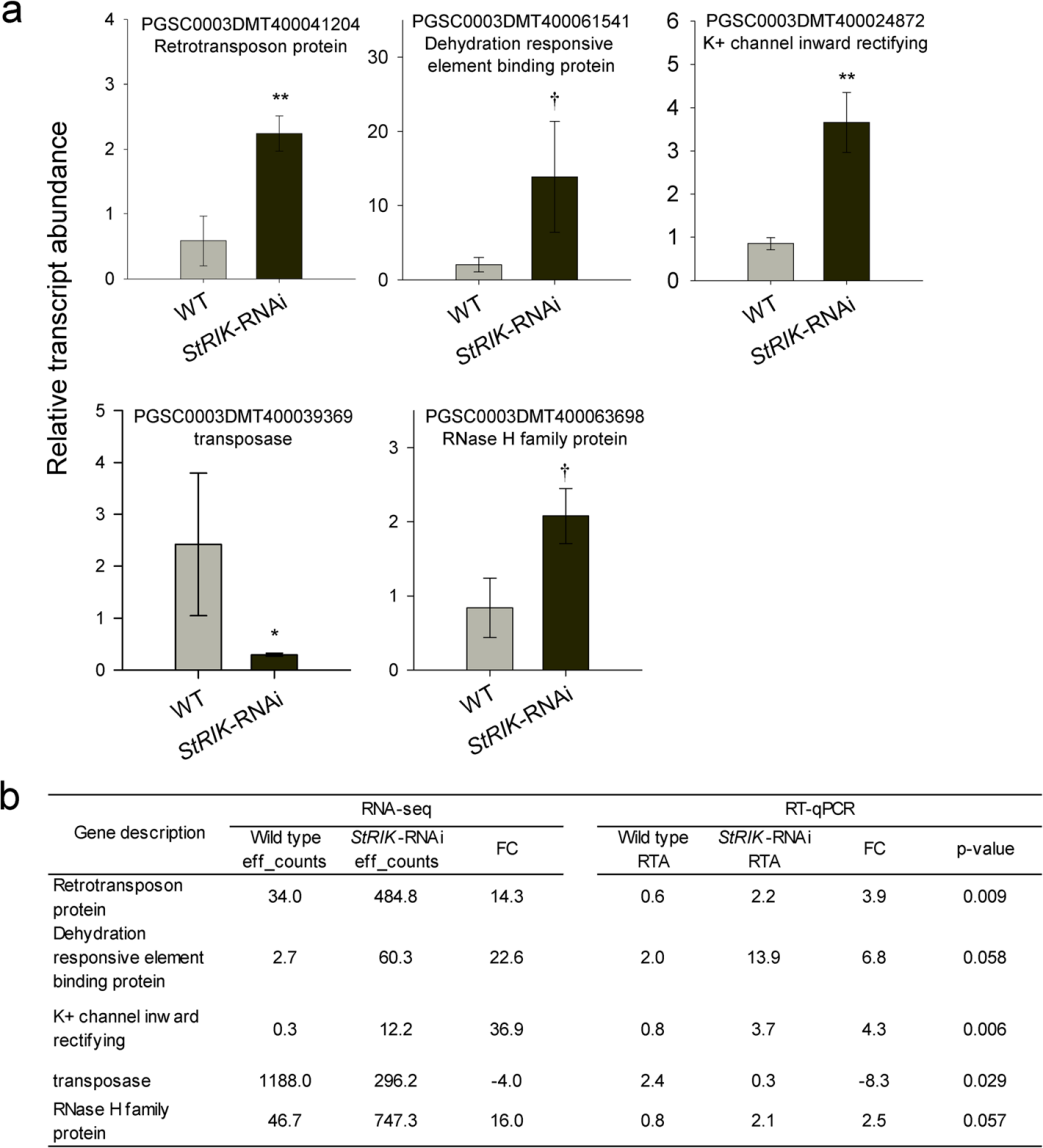
RT-qPCR analysis in *StRIK*-RNAi and Wild type tuber periderm of five DEGs. **a** The relative transcript abundance (RTA) of *Retrotransposon protein, Dehydration responsive element binding protein, K+ channel inward rectifying, transposase* and *RNase H family protein,* in StRIK deficient and Wild type lines is shown. Values are the mean ± SD of the Wild type (three biological replicates, *n* = 3) and *StRIK*-RNAi lines 12 and 47 (two biological replicates for each line, *n* = 4). The three lines were compared using a one-way analysis of variance with contrasts that showed no statistically significant difference between the two silenced lines for any of the five genes. However, after Benjamini-Hochberg adjustment for multiple testing, the difference between the mean of the two silenced lines and the wild type was statistically significant or of borderline significance for all five genes (two asterisks (**, *P* < 0.01), one asterisk (*, *P* < 0.05) and dagger (†, *P* < 0.06)). **b** Comparison of the results obtained for these genes in the RNA-seq and the Real-time PCR analyses. Results show the transcript abundance estimated by each method (effective counts for RNA-seq and RTA for RT-qPCR) as well as the log2 Fold Change (FC) obtained

To validate the RNA-seq results, the relative expression of five DEGs was analyzed by RT-qPCR in periderm from *StRIK*-RNAi (lines 12 and 47) and Wild type tubers grown at a different time to those used for the RNA-seq (Fig. 5a). The differential expression of the genes analyzed by RT-qPCR confirmed (Fig. 5a) the RNA-seq findings (Fig. 5b).

### The biological processes identified in the co-expression network of Arabidopsis *RIK* correlate with the transcriptome of *StRIK*-RNAi periderm

We explored the co-expression network of the Arabidopsis *RIK* gene by selecting the 300 genes most co-expressed with *RIK* protein based on ATTED-II (https://atted.jp/ [33];). Among the top 50 co-expressed genes, there were splicing factors (e.g. PWI domain-containing protein, *CC1*-like), two flowering time gene (*FCA*, *EDM2*), development genes (*TTL, cyclin-related*, *REV1*), other KH domain-containing proteins (e.g. At3g32940, At4g10070) and a microRNA (*MIR834a*) (Table S3). The gene ontology (GO) enrichment of these 300 co-expressed genes in the PlantGSEA database [34] highlighted processes related to RNA metabolism (e.g. ‘mRNA processing’ ‘RNA metabolic process’, ‘poly(A) RNA binding’), RNA splicing (e.g. ‘RNA splicing’, ‘mRNA splicing, via spliceosome’, ‘RNA splicing, via transesterification reactions’), regulation (e.g. ‘regulation of gene expression’, ‘regulation of biological process’), gene silencing (e.g. ‘gene silencing’, ‘gene silencing by RNA’) and development (e.g. ‘post-embryonic development’, ‘vegetative to reproductive phase transition of meristem’, ‘flower development’). Several GO terms related to the nucleus and spliceosome were identified within the cellular component classes (Table S4).

## Discussion

The potato *StRIK* gene, as well as its orthologues in Arabidopsis (At3g29390) and maize, are genes of unknown function. They are putative RNA-binding polypeptides with a K-homology (KH) domain. Our results showed *StRIK* ubiquitous expression in different plant tissues at similar levels (Fig. S2), which is in accordance with the *S. phureja RIK* RNA-seq data extracted from the PGSC [30] and with the transcript profile of its orthologue in maize [23]. Although Soler et al. [7] reported upregulation of the cork oak *QsRIK* gene in phellem compared with xylem (FC = 5), the present work suggests that the role of this potato gene is not specific to phellem or suberized tissues. Potato and Arabidopsis *RIK* genes are upregulated in flowers, fruits and in the shoot apex inflorescence during floral transition (Fig. S2). We found that *StRIK*-RNAi plants displayed floral transition in the shoot apical meristem and mostly formed fully developed flowers whereas Wild type plants did not, despite growing in parallel under the same conditions (Fig. 3a-d). This phenotype suggests that downregulation of *StRIK* could be required for organ specification or tissue differentiation and therefore a repressor role of *StRIK* in flower development could be hypothesized. Several proteins containing the K homology (KH) domain have been shown to affect flowering in Arabidopsis [35]. Specifically, the protein *HEN4* was involved in the pre-mRNA processing of the *AGAMOUS* floral homeotic gene [24]; *FLK* inactivation triggered *FLC* upregulation, possibly by modulating posttranscriptional gene regulation of *FLC* [25]; and the *PEPPER* KH-domain protein was shown to affect pistil development [27]. Its overexpression induced an increase of *FLC* transcript levels and a flowering delay, presumably by transcriptional and posttranscriptional regulatory mechanisms [36]. However, *S. phureja RIK* is induced in young growing tissues such as stolon and tuber sprout (Fig. S2). This and the ubiquitous gene expression (Fig. S2), suggest that *StRIK* plays a regulatory role in plant development other than flowering.

There is an upregulation of *StRIK* after wounding in potato tuber discs (Fig. S2). and the *S. phureja RIK* shows increased transcript accumulation upon osmotic stress (mannitol), salt stress, ABA treatment and during leaf senescence, but is repressed by heat treatment and wounding (24 h after wounding in leaves) (Fig. S2). In contrast, in Arabidopsis, wounding induces a mild *RIK* transcript accumulation in root 3, 6, 12 and 24 h after injury [37].

Similarly, the expression of genes involved in response to stress was altered when *StRIK* was silenced in the tuber periderm. Six genes whose Arabidopsis orthologues are involved in response to ABA and water deprivation (potato annotation: dehydrin, 11S globulin, dehydration responsive element binding protein, K+ channel inward rectifying, ribulose bisphosphate carboxylase large chain, vacuolar H + -pyrophosphatase, Table 1, Table S2, Fig. 5) were upregulated in *StRIK*-RNAi periderm. For instance, in Arabidopsis, the dehydration responsive element binding protein was related to drought, salt and heat stress responses [38], and the K-channel was involved in potassium cell homeostasis and ABA signal transduction [39, 40]. Conversely, other abiotic stress genes were downregulated, such as a wound responsive protein, a heat shock binding protein, a metallothionein and the LOB domain-containing protein 41, a transcription factor known to be induced by hypoxia in Arabidopsis [41] (Table 1, Table S2). Finally, three biotic stress genes were upregulated in these lines: one pathogenesis related and two orthologues to a member of Kunitz trypsin inhibitors (KTI) (At1g17860) (Table S2, stress and proteolysis categories), which play prominent roles in defense response against herbivores and in the response to wounding and methyl jasmonate [42–44]. It is worth to remark that biotic and abiotic stress response were identified in transcriptomic and proteomic approaches in potato tuber periderm [45, 46] and lately it was reported that ABA triggers suberin accumulation in the endodermis [47] and is relevant for periderm development [8]. Altogether suggests that StRIK could be significant for cork development related with biotic and abiotic stress signaling.

It is remarkable that most genes related to RNA metabolism are upregulated in *StRIK* silenced periderm. There are several RNase H proteins with unknown function in plants, but with pivotal roles in mammalian cell physiology and health, related to genome stability and cell viability [48, 49]. Other upregulated genes were a RNA-binding protein encoding for a chloroplast enzyme involved in rRNA maturation and intron recycling (At3g13740, [50]), a gene involved in splicing (At2g16860, [51]) and several rRNA intron-encoded homing endonucleases (Table 1, Fig. 5, Table S2). Accordingly, the co-expression network of the Arabidopsis *RIK* gene was enriched in several ontologies related to RNA metabolism and splicing (Table S3 and S4). The co-expressed genes included *FCA*, a controlling flowering gene [52] and a splicing factor *U2AF65A,* involved in intron recognition in plants [53, 54] and with capacity to regulate flowering time [55]. Also, RIK co-expressed with two KH-domain RNA binding proteins, SHINY and HOS5, which mediate correct pre-mRNA processing of stress-related genes under stress [28, 56]. Altogether, the DEGs related to stress and RNA metabolism and the co-expression network of Arabidopsis RIK suggest that *StRIK* could have a role in the periderm by interfering with the genome stability and/or mRNA maturation/stability, and stress signaling pathway.

Among the DEGs it is of note to mention several genes related to DNA transposition which were both up and downregulated in *StRIK*-RNAi lines (Table 1, Fig. 5 and Table S2). Transposable elements (TE) are mobile genetic elements abundant in genomes, which, upon activation, can alter gene expression [57] triggering effects in plant physiology, development or stress responses [58]. Because uncontrolled transposition is often deleterious, plants have evolved mechanisms to silence the transposons [59] through small interfering RNAs (siRNAs) responsible for RNA-directed DNA methylation (RdDM) [57]. Considering that RIK may bind regulatory RNAs [17] and that gene silencing is a process enriched in the *RIK* co-expression network (Table S4), it is tempting to speculate that StRIK may contribute to the epigenetic control of TEs during plant development and under stress conditions.

The role of StRIK in phellem remains unknown as its silencing does not affect phellem anatomy (Fig. 4) or the known suberin genes [60]. However, CYP87A2, that was downregulated in *StRIK*-RNAi periderm, was also identified as a phellem formation candidate because it is upregulated in cork compared with wood [7]. In addition, CASPL4D2, with unknown function, was also downregulated in *StRIK*-RNAi periderm (Table S2). Interestingly, CASPL4D1, which is very similar to CASPL4D2, is required for pathogen-induced lignification [61]. It is noteworthy that CASPL4C1, which is in the same gene subfamily as CASPL4D2 [62], showed earlier flowering and higher tolerance to cold stress when knocked out [63].

## Conclusions

Basing on the cork upregulation versus wood of the cork oak *RIK*, we focused on the function of the StRIK in the periderm by a reverse genetic approach in potato. Results showed that StRIK is encoded by a single gene in potato, as Arabidopsis and maize, contains two SF1-like K-homology RNA binding domains and displays a nuclear localization. The transcript accumulated in all the constitutive tissues and was induced by wounding in potato tuber. *StRIK* downregulation correlated with flower development solely in transgenic lines, while no evident changes in periderm anatomy were found. Nonetheless, transcriptome analysis highlighted 101 genes differentially expressed between *StRIK*-RNAi and Wild type periderm lines, which belong to functions related to RNA metabolism, stress, transposable elements and signaling. Altogether, results suggest that *StRIK* might play a regulatory role in potato tuber periderm through stress signaling and RNA metabolism.

## Methods

### Plant material

The potato plant cultivar (*S. tuberosum* Group Tuberosum cv. Désirée) was kindly provided by Professor Salomé Prat (Center for Research in Agricultural Genomics: CRAG, Barcelona, Spain). The tetraploid potato cultivar Désirée was obtained from crossing *Urgenta* x *Depesche* cultivars by ZPC breeder (Holland). The information of Désirée cultivar is available at the Potato Pedigree database (https://www.plantbreeding.wur.nl/PotatoPedigree/lookup.php?name=DESIREE:%20identifier%2011213, Wageningen University) and the European Cultivated Potato Database (ECPD: https://www.europotato.org/varieties/view/Desiree-E). Potato tuber periderm was used to isolate the *StRIK* full-length coding sequence, to produce the *StRIK* silenced plants to perform reverse transcription followed by quantitative PCR **(**RT-qPCR) and RNA-seq experiments. To obtain Désirée tubers*,* in vitro plants were propagated as described by Serra et al. [64] and then transferred to soil and grown for 3 months in a walk-in chamber before tuber harvest. The skin of potato tuber was manually dissected using sterile scalpels and was immediately frozen in liquid nitrogen. When the phellogen is active it is prone to break, hence the skin is easily removed and the tissue recovered contains mainly phellem but also phellogen. From now on, we will use the term periderm to refer to the potato skin harvested that contains phellem and phellogen. Potato tubers of *S. tuberosum* Group Tuberosum cv. Monalisa were purchased in a local supermarket and used to study the *StRIK* responsiveness to wounding. To that aim, potato tuber discs (3 mm thick and 13 mm in diameter) from flesh (parenchyma) were obtained with a cork borer and were left in a plastic box at room temperature, in darkness and saturated humidity conditions until sample harvesting.

### Cloning and sequencing the full-length of *StRIK*

For complete coding sequence isolation, first strand cDNA was synthesized using SuperScript III reverse transcriptase (Invitrogen, http://www.invitrogen.com/), oligo (dT)_18_ primer and total RNA from periderm tissue [65] previously treated with DNAse. The primers used to clone the full-length coding sequence of potato *StRIK* gene (Table S5) were designed based on the information from the potato Expressed Sequence Tag assembly (TC127409 and TC155463). PCR was performed using tuber periderm cDNA and the high fidelity PrimeSTAR® HS DNA Polymerase (Takara). PCR product was cloned into pCR4-TOPO (Invitrogen) and sequenced using BigDye [66] Terminator 3.1 kit (Applied Biosystems). The GenBank accession number of *StRIK* full-length coding sequence is MT622318.

### Potato transformation

The hairpin RNAi construct for *StRIK* gene silencing was obtained by PCR amplification (Table S5) of a specific fragment of 246 bp (Fig. S3). Amplification products were first cloned into pENTR/D-TOPO vector (Life Technologies) and then transferred in opposite orientations into the binary destination vector pBIN19RNAi [67] by LR clonase II enzyme (Life Technologies). Potato plant transformation was carried out as described by Fernández-Piñán et al. [66]. In brief, *A. tumefaciens* (GV2260) was transformed with the recombinant pBIN19RNAi vector and used to infect leaf explants, which were treated with phytohormones to induce the organogenesis process leading to kanamycin-resistant potato plants with the *StRIK* gene downregulated.

### Reverse transcription and quantitative PCR analysis (RT-qPCR)

Total RNA was isolated following the protocol reported by Logemann et al. [65]. First-strand cDNA was synthesized from 2 μg of DNAse digested RNA using the High Capacity cDNA Reverse Transcription kit (Applied Biosystems). Real time PCR analyses were performed in a LightCycler® 96 Real-Time PCR System (Roche). Gene-specific primers were designed with Primer3 0.4.0 software (https://bioinfo.ut.ee/primer3-0.4.0/) and then checked with NetPrimer (http://www.premierbiosoft.com/netprimer/). Each 20 μl qPCR reaction contained 10 μl of SYBR Green Select Master Mix (Applied Biosystems), 300 nM of each forward and reverse corresponding primer and 5 μl of a 100-fold diluted cDNA. The thermal cycle program used was a first step of 95 °C for 10 min and 40 cycles of 95 °C for 10 s, 60 °C for 15 s and 72 °C for 10 s. A dissociation final step was included to verify the presence of a single amplicon. For each primer pair, standard curves with a five-fold dilutions series of Wild type periderm cDNA template (1/5, 1/25, 1/125, 1/625 and 1/3125) was used to determine amplification efficiency, *E* = 10 ^(− 1/slope)^. The mRNA abundances for each gene were calculated as relative transcript abundance = (E_target_)^ΔCt target (control-sample)^ / (E_reference_)^ΔCt reference (control-sample)^ [68]. cDNA control sample for native tissues analysis was a pool with equal amounts of all samples, for wounding stress assay a pool of 144 h post-wounding replicates and for transgenic lines and RNA-seq validation, a pool of Wild type periderm replicates. The housekeeping gene *adenine phosphoribosyl transferase* (*APRT*) was used to normalize the results, except for the wounding experiment in which the constitutive gene *Elongation Factor 1 α* (*EF1α*) was used [69]. Gene-specific primer sequences are available in Table S5.

### Protein sequence alignment analysis

The amino acid multisequence alignment was performed using the Clustal Omega program from the European Bioinformatics Institute (EBI, https://www.ebi.ac.uk/Tools/msa/clustalo/). The alignment was edited using BOXSHADE version 3.21 available at https://embnet.vital-it.ch/software/BOX_form.html.

### Subcellular localization of RFP-StRIK fusion protein and periderm microscopy

*StRIK* gene coding region was amplified with specific primers bearing the attB recombinant sequences at 5′-end (Table S5) using PrimeSTAR® HS DNA Polymerase (Takara). The amplicon was cloned into the GATEWAY donor vector pDONR207™ (Life Technologies) and then transferred into the destination vector pK7WGR2.0 [70] to fuse the RFP to the N-terminal end of StRIK (pK7WGR2.0-*RIK*). *A. tumefaciens* cells (GV3101) transformed with the pK7WGR2.0-*RIK* vector and the HcPro silencing suppressor [71] were grown in parallel overnight at 28 °C in YEB liquid medium supplemented with the appropriate antibiotics. The cultures were centrifuged at 4000 g, the cell pellet resuspended in infiltration buffer (10 mM MES (pH 5.6), 10 mM MgCl_2_ and 500 μM acetosyringone) at 2 unit OD_600_/ml each culture and the mixture was incubated at room temperature for 2 h. Before agroinfiltration, both *Agrobacterium* samples (*RFP-RIK* and HcPro) were mixed in a 1:1 ratio to achieve 1 unit OD_600_/ml each culture. This mixture was used to agroinfiltrate the abaxial side of *N. benthamiana*. After 3 days, transformed cells were observed under a NIKON Ti Eclipse fluorescence inverted microscope with a confocal unit NIKON A1R. To detect red fluorescence, leaves were excited at 543.5 nm wavelength and the emission was collected at 595 nm. The software used for microscope imaging was NIKON NIS-Elements AR v 4.10. The scanning electron microscopy (SEM) was used to analyze the periderm anatomy as previously reported by Serra et al. [67] using 21-d stored tubers.

### RNA-seq high-throughput sequencing

Periderm (skin) was isolated from freshly harvested potato tubers avoiding the underlying cortical parenchyma (see Plant Material subsection). Total RNA was purified by means of the PureLink® Plant RNA Reagent (Ambion) using a modification of the standard protocol by repeating step four and five of the protocol twice and adding a KOAc 2 M (pH 5.5) precipitation step to remove polysaccharides. Final RNA precipitation was performed with the GlycoBlue™ Coprecipitant (Ambion). Genomic DNA was removed using TURBO DNA-free kit (Ambion) according to the manufacturer’s instructions. RNA samples were analyzed using Agilent 2100 Bioanalyzer and those with an RNA integrity number (RIN) over 7 were sequenced. Three biological replicates were sequenced for each line (Wild type and *StRIK*-RNAi lines 9, 12 and 47). The cDNA libraries were prepared using the TruSeq RNA Library Prep Kit (Illumina) following the manufacturer’s protocols and then ran in an Illumina HiSeq 2000 instrument (BGI Hong Kong). The quality of the RNA-seq data was analyzed using FastQC v0.11.2 (https://www.bioinformatics.babraham.ac.uk/projects/fastqc/). The reads were aligned with Bowtie 2 [72] against the *S. tuberosum* Group Phureja transcriptome generated from the genome assembly v4.03 [30] using the most recent version of the GFF3 genome annotation file (PGSC_DM_V403_genes.gff.zip) with the program gffread from the Cufflinks package [73]. The quantification of transcript abundance was performed with eXpress 1.5.1 [74]. The column labelled ‘eff_counts’ obtained from the eXpress output was passed as input to baySeq [75] for the differential expression analysis. For each model fitted, transcripts with a False Discovery Rate (FDR) less than 0.05 were considered differentially expressed. All sequencing data are available in the Gene Expression Omnibus repository from NCBI under accession code GSE153641.

## Supplementary Information

**Additional file 1: Fig. S1** Amino acid alignment of the potato (*S. tuberosum* Group Tuberosum) StRIK protein with the most homologous proteins of *S. tuberosum* Group Phureja (PGSC0003DMP400043638), *S. lycopersicum* and *S. pennellii* (XP_004233384.1 and XP_015065578.1)*,* Arabidopsis (AAY24687.1) and maize (AAY24682.1). The Arabidopsis splicing factor 1 SF1-like, At5g51300, identified by Lorkovic and Barta (2002) as KH domain protein, and the two most homologous proteins in potato PGSC0003DMP40003285 and PGSC0003DMP400012836 (designated as S.tubPhurSF1_1 and S.tubPhurSF1_2, respectively) are also included. The two predicted SF1_like-KH conserved protein domains and the highly conserved KH domain core consensus sequence IIGxxGxxI described by Burd and Dreyfuss [21] are indicated. The proline-rich region identified is also shown. The amino acids that are identical are shaded in black and the ones that are similar in grey. The following abbreviations were used for the RIK and SF1 sequences: S.tub, *S. tuberosum* Group Tuberosum; S.tubPhur, *S. tuberosum* Group Phureja; S.lyc, *S. lycopersicum*; S.pen, *S. pennellii*; Arab, Arabidopsis; Z.may, *Z. mays.*

**Additional file 2: Fig. S2** Transcript accumulation profile of *StRIK and S. phureja RIK* in different tissues and conditions. (a) Relative transcript abundance (RTA) of *StRIK* accumulation in different potato organs and tissues, RTA levels were expressed as the mean ± SD of three technical replicates. (b) Transcript profile of *RIK* in *S. phureja* (DM and RH genotypes)*.* The FPKM values were obtained from data reported by the Potato Genome Sequencing Consortium [30] and Massa et al. (2011). A red-green colour gradient from lower and higher transcript accumulation is shown, respectively. (c) Accumulation of *StRIK* transcripts in potato tuber healing discs over 144 h. RTA levels are represented as the mean ± SD of two biological replicates. There was a highly significant linear increase in *StRIK* levels after wounding (regression analysis to test linear relationship between transcript abundance and time: *p* < 0.001).

**Additional file 3: Fig. S3** Genomic *StRIK* sequence. Nucleotide sequence used for *StRIK* silencing is shown in red characters. The 12 exons of *StRIK* gene are shadowed in grey. The start initiation and the STOP codons are highlighted in green.

**Additional file 4: Fig. S4** Target region of the 246 bp *StRIK-*RNAi fragment underlined in grey on the *RIK* gene (PGSC0003DMG400025145) genomic sequence visualized in the Spub DB Genome Browser (http://solanaceae.plantbiology.msu.edu/cgi-bin/gbrowse/potato/) from the [30]. The *RIK* gene is encoded in chromosome 2 antisense strand (−) between 25,304,888-25,313,841 bp positions in the *S. tuberosum* Group Phureja and it is composed of 12 exons and 11 introns which transcribes for four predicted gene isoforms. The *StRIK*-RNAi fragment targets exon 8, 9 and 10 from the representative transcript (PGSC0003DMT400064730) as well as two other gene isoforms PGSC0003DMT400064729 and PGSC0003DMT400064731 while the fourth and shortest isoform PGSC0003DMT400064735 is not targeted by the RNAi fragment.

**Additional file 5: Fig. S5***StRIK* transcript accumulation measured by RT-qPCR in potato a) leaf and b) tuber periderm of *StRIK*-RNAi and Wild type lines from two consecutive plantings. Each column represents the mean and standard deviation of three technical replicates. Lines selected in leaves for further testing in tuber periderm are indicated with a red arrow.

**Additional file 6: Table S1** BLASTN results against potato transcript database with the *StRIK*-RNAi construct as a query.

**Additional file 7: Table S2** RNA-seq results of genes showing differential expression between Wild type and *StRIK*-RNAi potato tuber periderm (FDR < 0.05). Gene functional annotation of potato and Arabidopsis (e value ≤10  ^− 5^), gene expression levels (effective counts) and log_2_FC values (*StRIK*-RNAi/WT) are shown.

**Additional file 8: Table S3** List of the 300 most co-expressed genes with Arabidopsis *RIK* gene based on ATTED-II database (https://atted.jp/). Co-expression degree is represented as Mutual Rank (MR) value.

**Additional file 9: Table S4** Gene ontology of 300 most co-expressed genes with *RIK* gene in Arabidopsis by PlantGSEA web tool (http://structuralbiology.cau.edu.cn/PlantGSEA/index.php).

**Additional file 10: Table S5** List of primers used.

## Data Availability

The StRIK full-length coding sequence is in the GenBank: accession number MT622318 (https://www.ncbi.nlm.nih.gov/genbank/). The RNA-seq data generated during the current study have been deposited in the Gene Expression Omnibus repository from NCBI under accession code GSE153641 (https://www.ncbi.nlm.nih.gov/geo/query/acc.cgi?acc=GSE153641). Databases and programs used in this study are the following: Arabidopsis Information Resource (https://www.arabidopsis.org/), Spud DB Potato Genomics Resources (http://solanaceae.plantbiology.msu.edu/pgsc_download.shtml), ATTED-II (https://atted.jp/) Plant GeneSet Enrichment Analysis Toolkit database (http://structuralbiology.cau.edu.cn/PlantGSEA/index.php) FastQC v0.11.2 (https://www.bioinformatics.babraham.ac.uk/projects/fastqc/), Clustal Omega program from the European Bioinformatics Institute (EBI, https://www.ebi.ac.uk/Tools/msa/clustalo/), BOXSHADE version 3.21 (https://embnet.vital-it.ch/software/BOX_form.html), Primer3 0.4.0 software (https://bioinfo.ut.ee/primer3-0.4.0/), NetPrimer (http://www.premierbiosoft.com/netprimer/), Potato Pedigree database (https://www.plantbreeding.wur.nl/PotatoPedigree/lookup.php?name=DESIREE:%20identifier%2011213) and the European Cultivated Potato Database (ECPD: https://www.europotato.org/varieties/view/Desiree-E). Public access to all these databases is open. The material used in this study will be shared on reasonable request to the corresponding author.

## Abbreviations

AS1: Asymmetric leaves 1
DEGs: Differentially expressed genes
GO: Gene ontology
KH: K-homology
rs2: Rough sheath2
RIK: Rs2-interacting KH protein
SF1: Splicing factor 1
RFP: Red fluorescent protein
