# Correction: Silencing of StRIK in potato suggests a role in periderm related to RNA processing and stress

**DOI:** 10.1186/s12870-022-03994-y

**Published:** 2022-12-24

**Authors:** Pau Boher, Marçal Soler, Sandra Fernández-Piñán, Xènia Torrent, Sebastian Y. Müller, Krystyna A. Kelly, Olga Serra, Mercè Figueras

**Affiliations:** 1 Laboratori del Suro, Biology Department, Universitat de Girona, Campus Montilivi, E-17071 Girona, Catalonia Spain; 2 Department of Plant Sciences, University of Cambridge, Downing Street, Cambridge, CB2 3EA UK

**Correction:** ***BMC Plant Biol*** **21, 409 (2021)**

**https://doi.org/10.1186/s12870-021-03141-z**

Following the publication of the original article [1], the author requested for an updated in the Funding section of this article. Below funding reference should be added:

- PID2019-110330GB-C21 (MCI/AEI/FEDER, UE)

The correction does not have any effect on the results or conclusions of the paper. The original article has been corrected.
