# Supplementary material for: Silencing of StRIK in potato suggests a role in periderm related to RNA processing and stress
Source: BMC Plant Biol. 2021 Sep 7;21:409. doi: 10.1186/s12870-021-03141-z (PMC8424952; doi:10.1186/s12870-021-03141-z)
Supplement: Supplementary file 1 — Additional file 1: Fig. S1 Amino acid alignment of the potato (S. tuberosum Group Tuberosum) StRIK protein with the most homologous proteins of S. tuberosum Group Phureja (PGSC0003DMP400043638), S. lycopersicum and S. pennellii (XP_004233384.1 and XP_015065578.1), Arabidopsis (AAY24687.1) and maize (AAY24682.1). The Arabidopsis splicing factor 1 SF1-like, At5g51300, identified by Lorkovic and Barta (2002) as KH domain protein, and the two most homologous proteins in potato PGSC0003DMP40003285 and PGSC0003DMP400012836 (designated as S.tubPhurSF1_1 and S.tubPhurSF1_2, respectively) are also included. The two predicted SF1_like-KH conserved protein domains and the highly conserved KH domain core consensus sequence IIGxxGxxI described by Burd and Dreyfuss [21] are indicated. The proline-rich region identified is also shown. The amino acids that are identical are shaded in black and the ones that are similar in grey. The following abbreviations were used for the RIK and SF1 sequences: S.tub, S. tuberosum Group Tuberosum; S.tubPhur, S. tuberosum Group Phureja; S.lyc, S. lycopersicum; S.pen, S. pennellii; Arab, Arabidopsis; Z.may, Z. mays. [file 12870_2021_3141_MOESM1_ESM.docx]

S.tubRIK 1 -----------------------------------------------------------------------------------------MTEDNCP-RVS----SSETVD
S.tubPhurRIK 1 -----------------------------------------------------------------------------------------MTEDNCP-RVS----SSETVD
S.lycRIK 1 -----------------------------------------------------------------------------------------MTEDNCP-RVS----SSETVD
S.penRIK 1 -----------------------------------------------------------------------------------------MTEDNCP-RVS----SSETVD
ArabRIK 1 -----------------------------------------------------------------------------------------MTEDNDEARVP---LSDSSTT
Z.mayRIK 1 -----------------------------------------------------------------------------------------MTEDRAHKV-------ADEPA
AT5G51300SF1 1 --------------MESVE--------MNNPNSQTLDQPPPSSNGDTAPLALDHMNPQNSESVALNGSS-TPIPDTNGSSAKPELLRPLLSENGVSKTLSG-NDKDQSGG
S.tubPhurSF1_1 1 MD------------------YQSQSPSV-----ETLDHNSSQN---QA-----SYDAQLHASDNTGPNQASEEQKLGEFTLKREIQRPLLSENGLTNTHSG-TDRDQSGG
S.tubPhurSF1_2 1 MDSQSHPVQEPSQTVNSYEQYQNSSDSYYQNPSQTLGQDPAPGVCENAEL--NGSNSNFRENSAKPGDNNSAQNKLSDLNSNLMLQKPLLSGNGLTNTHSGGAERDQSGG


S.tubRIK 17 SNSSSTKQRKRRKWDQPAETFVPEGVAVS--GIFPLV--------NTGSLAGITLPGVIPVLGAAFTNPLI------AIGATTVQQLPVIIAQKSVQPKIQDE-LIAREI
S.tubPhurRIK 17 SNSSSTKQRKRRKWDQPAETFVPEGVAVS--GIFPLV--------NTGSLAGITLPGVIPVLGAAFTNPLT------AIGATTVQQLPVIIAQKSVQPKIQDE-LIAREI
S.lycRIK 17 SNSSSTKQRKRRKWDQPAETFVPEGVAVS--GIFPLA--------NTGSLAGITLPGVIPVLGAAFTNPLT------TIGATTVQQLPVIIAQKSVQPKIQDE-LIAREI
S.penRIK 17 SNSSSTKQRKRRKWDQPAETFVPEGVAVS--GIFPLA--------NTGSLAGITLPGVMPVLGAAFTNPLT------AIGATTVQQLPVIIAQKSVQPKIQDE-LIAREI
ArabRIK 19 NDASRTRQRRKRKWDKPAEQLVAAGVAFP--QLLPLG--------NTMNVPS-----MSPLLQTL--------------------SVP-LAVPKVNQPKIQDEVIIAREI
Z.mayRIK 15 ASGRQSPERKKRKWDQPAEDLVSAAVTAAAVSGMPVM--------NFGALPGVVLPGVTAYGAATLPSVVPVPYSLPPHIAPSVLQNAAAAAQKLSQAKIPDE-VIAREI
AT5G51300SF1 87 EEETTSRRKRRSRWDPPPSESINNPSAEGGTDSGTGTRKRKSRWADDEPRTQIQLPDFMKDF----TGGI------------------------EFDPEIQAL--NSRLL
S.tubPhurSF1_1 79 EEETSSRRRRRSRWDPPPTESTNDG--TGGGDGSGTGRKRKSRWADDEPKPVIQLPDFMKDF----TGGI------------------------EFDPEIQAL--NSRLL
S.tubPhurSF1_2 109 EEETSSRRRRRSRWDPPPTDSSNDG--TGGNDGTGAGRKRKSRWADDEPKPVIQLPDFMKDF----AGGI------------------------EFDPEVQAL--NSRLL


S.tubRIK 110 VINDADPSVRYRLTKRQTQEEIQKSTGAVVITRGKYKPPSAPSDGEKPLYLHISAGAHL--ETTLERIRAVDRAAAVVEEMLKQSPVN---------NGLKVNHLLSTCV
S.tubPhurRIK 110 VINDADPSVRYRLTKRQTQEEIQKSTGAVVITRGKYKPPSAPSDGEKPLYLHISAGAHL--ETTLERIRAVDRAAAVVEEMLKQSPVN---------NGLKVNHLLSTCV
S.lycRIK 110 VINDADPSVRYRLTKRQTQEEIQKSTGAVVITRGKYKPPSAPSDGEKPLYLHISAGAHL--ETTLERIRAVDRAAAVVEEMLKQGPVN---------NGLKVNHLLSTCV
S.penRIK 110 VINDADPSVRYRLTKRQTQEEIQKSTGAVVITRGKYKPPSAPSDGEKPLYLHISAGAHL--ETTLERIRAVDRAAAVVEEMLKQGPVN---------NGLKVNHLLSTCV
ArabRIK 93 VINDAEASLRHRLTKRSTQEDIQRSTGAVVITRGKYRPPNAPPDGEKPLYLHISAAAQLQLKETTERILAVDRAAAMIEEMMKQKSISQIGS-----VGLQTVKMLSTCV
Z.mayRIK 116 VINDADPSVRYKLTKRQTQEEIQKCTNTVIITRGKYHPPNLLPDGEKPLYLHISAGSQL--KDTAERIKAVDRAASMIEEILKQGTTSESISVPFSSSTGQAVRPFSASV
AT5G51300SF1 167 EI------------SRMLQS------GMP----LDDRPEGQRSPSPEPVYDNMGIRINT--REYRARERLNRERQEIIAQIIKKNPAF-------KPPADYRPPKLHKKL
S.tubPhurSF1_1 157 EI------------SRKLQS------GMP----LDDRPDGARSPSPEPIYDNMGIRINT--REYRAREKLNRERQEIISQIIKKNPAF-------KPPADYRPPKLQKKL
S.tubPhurSF1_2 187 EI------------SRKLQS------GMP----LDDRPEGARSPSPEPIYDNMGVRINT--REYRAREKLNRERQEIISQIIKKNPAF-------KPPADYRPPKLHKKL


S.tubRIK 209 YLGFEADPSANITVRIRGPNDQYINHIMNETGATVLLRGRGSGYSDEGE-------GEDVYQPLHLLISSNNSASLERAKLLAENLLDTICAECGASRVS----------
S.tubPhurRIK 209 YLGFEADPSANITVRIRGPNDQYINHIMNETGATVLLRGRGSGYSDEGE-------GEDVHQPLHLLISSNNSASLERAKLLAENLLDTICAECGASRVS----------
S.lycRIK 209 YLGFEADPSANITVRIRGPNDQYINHIMNETGATVLLRGRGSGYSDEGQ-------GEDVHQPLHLLISSNNSASLERAKLLAENLLDTICAECGASRVS----------
S.penRIK 209 YLGFETDPSANITVRIRGPNDQYINHIMNETGATVLLRGRGSGYSDEGQ-------GEDVHQPLHLLISSNNSASLERAKLLAENLLDTICAECGASRVS----------
ArabRIK 198 YLGFEADPSSNVAARIRGPNDQYINHIMNETGATVVLRGRGSGSLENQH-------GDEAQLPLHLLLSGSNPKSIDDAKRLAENLMDTISVEFGASRVS----------
Z.mayRIK 224 FLGFDADPSLNITARIRGPNDQYINHIMKETGVTVVLRGKDSENLGSCH-------SEASQQPLHLYLTSMHLKNLEAAKVLAENLLDTVAAEFGASRIS----------
AT5G51300SF1 246 FIPMKEFPGYNFIGLIIGPRGNTQKRMERETGAKIVIRGKGSVKEGRHQQKKDLKYDPSENEDLHVLVEAETQEALEAAAGMVEKLLQPVDEVLNEHKRQQLRELATLNG
S.tubPhurSF1_1 236 YIPMKEFPGYNFIGLIIGPRGNTQKRMEKETGAKIVIRGKGSIKEGRFQQKGNLKPDPAENEDLHVLVEAENQESLEGAAAMIEKLLQPVDEVLNEHKRQQLKELAALNG
S.tubPhurSF1_2 266 YIPMKEYPGYNFIGLIIGPRGNTQKRMEKETGAKIVIRGKGSIKEGRFQQKGNLKHDPAENEDLHVLVEADTQESLEAAAAMLEKLLQPVDEVLNEHKRQQLRELAALNG


S.tubRIK 302 --------------------------------SCKVYGAVPPPLQ--PLASVQVSGSESEVNNIPTANVAAQIL-SFSTAA-AVPMTAA----------AGVTGVVSQGT
S.tubPhurRIK 302 --------------------------------SCKVYGAVPPPLQ--PLASVQVSGSESEVNNIPTANVAAQIL-SFSTAA-AVPVTAA----------AGVTGVVSQGT
S.lycRIK 302 --------------------------------SCKVYGAVPPPLQ--PLASVQVSGSESEVNNTPTANVAAQIL-SSSTAA-AVPVTAA----------AGGTGVVSQGT
S.penRIK 302 --------------------------------SCKVYGAVPPPLQ--PLASVQVSGSESEVNNTPTANVAAQIL-SSSTAA-AVPVTAA----------AGGTGVASQGT
ArabRIK 291 --------------------------------SSKVYGAVPPPQQ--LISGAPGSDQE--NQNLI--------S-TYGLMT-SIPITAPPYAVSSFPV-TPATSLYPQFP
Z.mayRIK 317 --------------------------------SSKVYGAVPPPQQ--LLAGVDTSGTKSDVHYIVGPNVLSGATHSFASTGVIAPVVAPAV------------------T
AT5G51300SF1 356 TIRDEEFCRLCGEPGHRQYACPSRTNTFKSDVLCKICGDGGHPTIDCPVKGTTGKKMDDEYQNFL-----A-------ELGGTVPESSLKQS-ATLALGPGSSGSNPPWA
S.tubPhurSF1_1 346 TIRDEEFCRLCGEPGHRQYACPSRTTTFKSDVLCKICGDGGHPTIDCPVKNTTGKKMDDEYQNFL-----A-------ELGGTIPESLTKQNPAALALGAGNSGSNPPWA
S.tubPhurSF1_2 376 TIRDEEFCRLCGEPGHRQYACPSRTTTFKSDVLCKICGDGGHPTIDCPVKNTTGKKMDDEYQNFL-----A-------ELGGTVPESSLKQNAATLALGPGSTGSNPPWA


S.tubRIK 366 ---VPQSLGSLNPVPSQPPT-SCYPHQLVTSRTSYIGYDGIYPQATALQQVALALRQSTSPVTTTVPPATTGPSITS--QTSTGTEKDRRPAQKRKFQELPAGGKGQSTV
S.tubPhurRIK 366 ---VPQSLGSLNPVPSQPPT-SCYPHQLVASRTSYIGYDGIYPQATALQQVALALRQSTSPVTTTVPPATTGPSITS--QTSTGTEKDKRPAQKRKFQELPAGGKGQSTV
S.lycRIK 366 ---VPQSLGSLDPVPSQPPT-SCYPHQLVTSRTSYIGYDGIYPQATALQQVALALRQSTSPVTSTVPPATTGPSITS--QTSTGSEKDKRPAQKRKFQELPAGGKGQATV
S.penRIK 366 ---VPQSLGSLDPVPSQPPT-SCYPHQLVTSRTSYIGYDGIYPQATALQQVALALRQSTSPVTSTVPPATTGPSITS--QTSTGSEKDKRPAQKRKFQELPAVGKGQATV
ArabRIK 354 ---VMQSLGISNG----------GPSQPVAGGTSYSGYAGIYPQATPLQQVAQVLKQSISPVISTVPPTMLTATSLSIPSDNASNEMERRPPRKRKFQELPADCKVPEKD
Z.mayRIK 375 ---VQSGAPTYSGV-PLPSN-MAYPIPPANGGAFYSGYGDIYPQATPLQQLAFTLKHASSSATQAVPVTSTPTSMATKGNSILDAEMDK--RSRRKFQELPVS-KGPATE
AT5G51300SF1 453 NN---AGNGAS-AHPGLGSTPTKPPSKEYDETNLYIGFLPPMLEDDGLINLF-------SSFGEIV--------------------MAK--VIKDRVTGL---SKGYGFV
S.tubPhurSF1_1 444 SNNNTGGAGAS-SHPGLGSSILKP--KEFDETNLYIGYLPPTLEDDGLINLF-------SHIGTIV--------------------MAK--VIKDRLSGL---SKGYGFV
S.tubPhurSF1_2 474 SSNNTSGGGTT-SHPGLGSNIMKP--KEFDDTNLYIGYLPPTLDDDGLINLF-------SPFGTIV--------------------MAK--VIKDRLSGL---SKGYGFV


S.tubRIK 470 NQNPLQGTEL---LMLQERMSD---------KGDRDKIG--TPAP-------------------------------RKLV----------------------QPLSSSML
S.tubPhurRIK 470 NQNPLQGTEL---LMLQERMSD---------KGDRDKIG--TPAP-------------------------------RKLV----------------------QPLSSSML
S.lycRIK 470 NQNPLQATEL---LMLQERISE---------KGDTDKIG--IPTP-------------------------------RKLV----------------------QPLSSSML
S.penRIK 470 NQNPLQAMEL---LMLQERISE---------KGDTDKIG--IPTH-------------------------------RKLV----------------------QPLSSSML
ArabRIK 451 K----QQSEL---AMTGDVTPSANR-VRSP-PSPRSVMP--PPPP-------------------------------KTIAPP--------------PSKTMSPPSSKSML
Z.mayRIK 477 SQNSQQGSKF---VKTGLDSSGNIG-SSSI-APPK-KV---HPGS-------------------------------NGMLPQEEADMPSHL----SISTKMLPPPLKSML
AT5G51300SF1 527 KYADVQMANTAVQAMNGYRFEGRTLAVRIAGKSPPPIAPPGPPAPQPPTQGYPPSNQPPGAYPSQQYATGGY----------STAPVPWGPPVPS-YSPYALPPPPPGSY
S.tubPhurSF1_1 519 KFADVQQANSAITSMNGHRLDGRTIAVRVAGKPPQPAVPPG-PAPAMP--SYPVPNQSMGAYPSQRYAAGGPIGNPPGSYPPPGAPVPWGPPVPPPYAQYPPPPPGAAMY
S.tubPhurSF1_2 549 KYSDVQQANSAIAGMNGHCLDGRTIAVRVAGKPPQPAVPPSPPAPAMP--PYPAPNQASGVYPSQQYATGGPIGPPGGY---AGTPVPWGPPVPPPYASYPPP--GSIMY


S.tubRIK 513 PPPPPRMM-------PPPPPPPKFQSSS--Q-----KVHDNNVVNKAPCKIVP----------DTLVQLMEYGDDDD-DDNDEAIDG-----------------------
S.tubPhurRIK 513 PPPPPRMM-------PPLPPPPKFQSSS--Q-----KVHDNNVVNKAPCKIVP----------DTLVQLMEYGDDDD-DDNDEAIDG-----------------------
S.lycRIK 513 PPPPPRMM-------PPPPPPPKFQSSS--Q-----KVHDNNMVNKAPSKIVP----------DTLVQLMEYGDDDDDDDNDEAIDG-----------------------
S.penRIK 513 PPPPPRMM-------PPPPPPPKFQSSS--Q-----KVHDNNVVDKAPSKIVP----------DTLVQLMEYGDDDD-DDNDEAIDG-----------------------
ArabRIK 505 PPPPRSKTMSPLSSKSMLPPPPRFTLTTQRS-----RLQDNHISV-KKPNPVP----------DTLIKLMEYGDDEDDDDDPD---E-----------------------
Z.mayRIK 543 PLPPRSMP--PPPPKSMPPPPPKFPSDEFLS-----RNENKFFPLKEPT-APP----------RSFDAISVLPSERRPREPKEEKNK-----------------------
AT5G51300SF1 626 -HPVHGQHMPPYGMQ-YPPPPPHVTQAPPPGTT-------QNPSSSEPQQSFPPGVQADSGAATSSIPPNVYGSSVTAMPGQP------PYMSYPSYYNAVPPPTPPA--
S.tubPhurSF1_1 626 P-PVAGQPIPPYGMQ-YPPSIPAASSGAPAQTVSSGENQQTYTSPGEAQQNYPPGMQSQ----------SVYGNSVKAMPPNAQPAYPTSSYSYPSYYGVTPPPP-PPSA
S.tubPhurSF1_2 652 PPPPPGQFVPPYGAQ-YPPPMPTPSSGVPAQTVSSGENQQNYTSSGETQQSYPPGVQSHNSAPVQSLPSYAYGNSVAALPPHTQPAYPTSSYSYPSYYGMAPPPPLPPTA


S.tubRIK 575 PLKSSSSAVATPKPFWAV*---------------------------------------------------------------------
S.tubPhurRIK 575 PLKCSSSAVATPKPFWAV*---------------------------------------------------------------------
S.lycRIK 576 PLKSSSSAVATPKPFWAV----------------------------------------------------------------------
S.penRIK 575 PLKSSSSAVATPKPFWAV----------------------------------------------------------------------
ArabRIK 573 PLTTRS----------------------------------------------------------------------------------
Z.mayRIK 612 RHTCV-----------------------------------------------------------------------------------
AT5G51300SF1 719 PASSTDHSQNMGNMPWANNPSVSTPDHSQGLVNAPWAPNPPMPPTVGYSQSMGNVPWAPKPPVQPPAENPSSVGESEYEKFMAEMK--
S.tubPhurSF1_1 723 SQSNGNYSQGMSNVPWA--------------------PNPPTHA------------------PPSSAEKPAYGADAEYEKFMSEMKS*
S.tubPhurSF1_2 761 TQSSVDHSQSMSNVPWA--------------------SNPPEPAP------------APPPPPPPSAEKPPYGTDAEYEKFMSEMK*-

**Proline-rich region (498-527 aa)**

**SF1-like KH (127-203 aa)**

**SF1-like KH (216-291 aa)**

**IIGxxGxxI**
