# Supplementary figures and images for: Silencing of StRIK in potato suggests a role in periderm related to RNA processing and stress
Source: BMC Plant Biol. 2021 Sep 7;21:409. doi: 10.1186/s12870-021-03141-z (PMC8424952; doi:10.1186/s12870-021-03141-z)

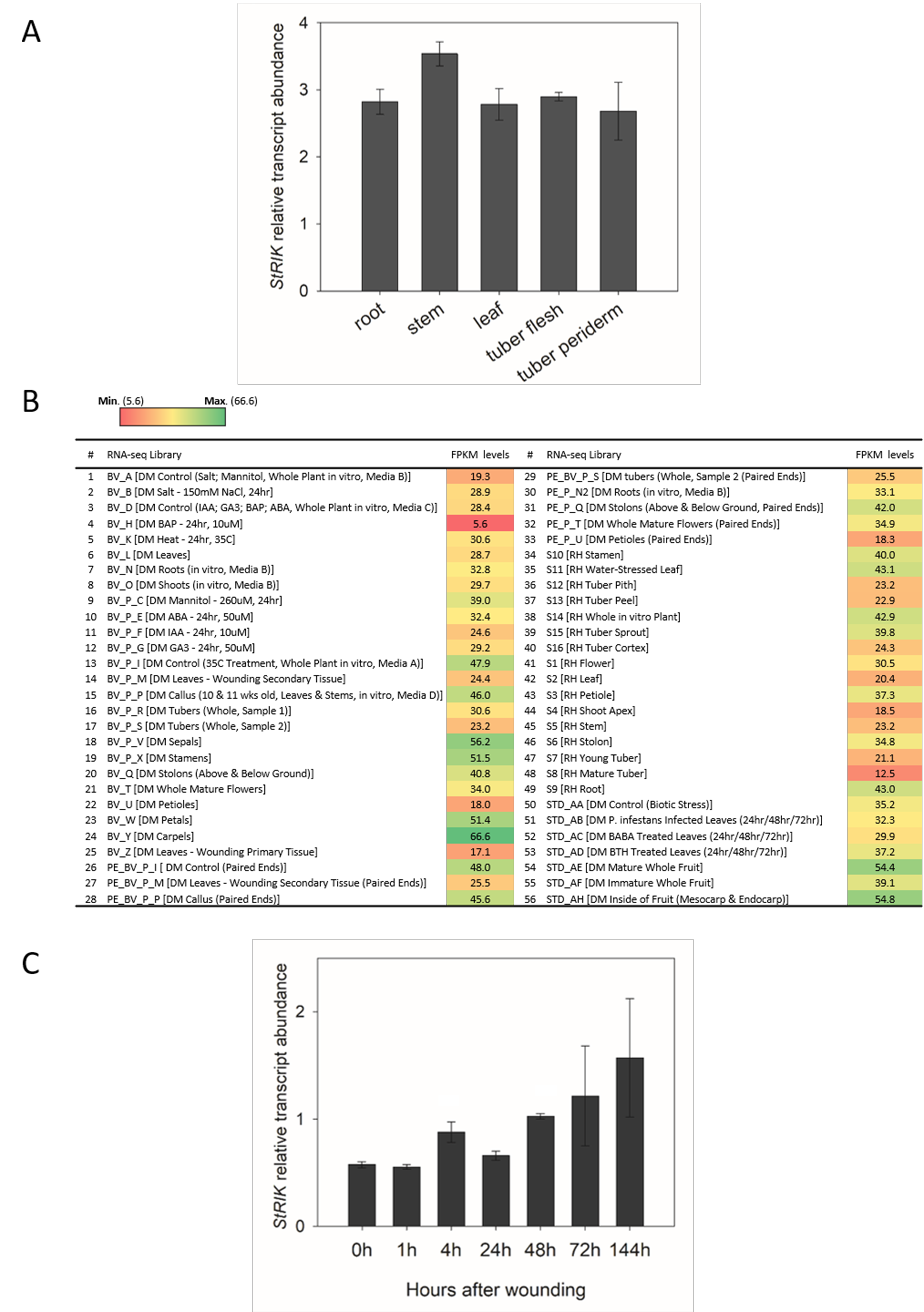

Supplement: Supplementary file 2 — Additional file 2: Fig. S2 Transcript accumulation profile of StRIK and S. phureja RIK in different tissues and conditions. (a) Relative transcript abundance (RTA) of StRIK accumulation in different potato organs and tissues, RTA levels were expressed as the mean ± SD of three technical replicates. (b) Transcript profile of RIK in S. phureja (DM and RH genotypes). The FPKM values were obtained from data reported by the Potato Genome Sequencing Consortium [30] and Massa et al. (2011). A red-green colour gradient from lower and higher transcript accumulation is shown, respectively. (c) Accumulation of StRIK transcripts in potato tuber healing discs over 144 h. RTA levels are represented as the mean ± SD of two biological replicates. There was a highly significant linear increase in StRIK levels after wounding (regression analysis to test linear relationship between transcript abundance and time: p < 0.001). [file 12870_2021_3141_MOESM2_ESM.tif]

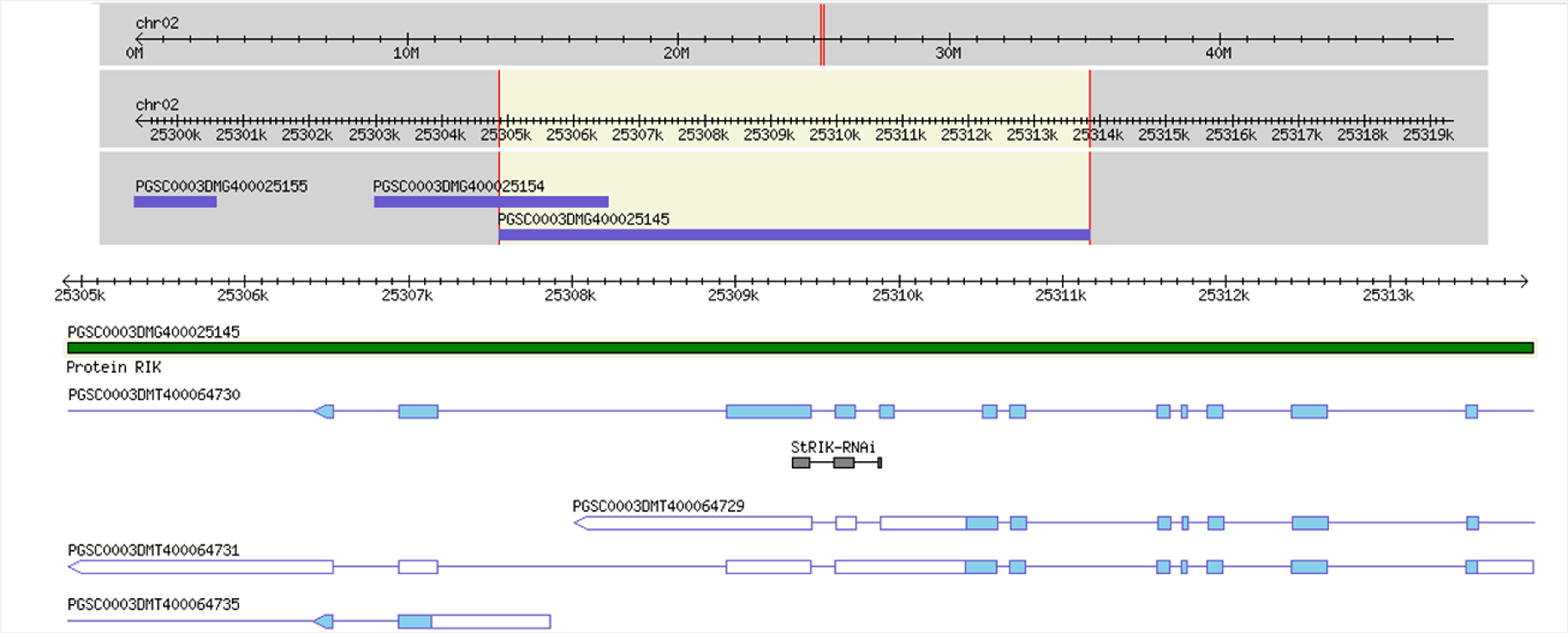

Supplement: Supplementary file 4 — Additional file 4: Fig. S4 Target region of the 246 bp StRIK-RNAi fragment underlined in grey on the RIK gene (PGSC0003DMG400025145) genomic sequence visualized in the Spub DB Genome Browser (http://solanaceae.plantbiology.msu.edu/cgi-bin/gbrowse/potato/) from the [30]. The RIK gene is encoded in chromosome 2 antisense strand (−) between 25,304,888-25,313,841 bp positions in the S. tuberosum Group Phureja and it is composed of 12 exons and 11 introns which transcribes for four predicted gene isoforms. The StRIK-RNAi fragment targets exon 8, 9 and 10 from the representative transcript (PGSC0003DMT400064730) as well as two other gene isoforms PGSC0003DMT400064729 and PGSC0003DMT400064731 while the fourth and shortest isoform PGSC0003DMT400064735 is not targeted by the RNAi fragment. [file 12870_2021_3141_MOESM4_ESM.tif]

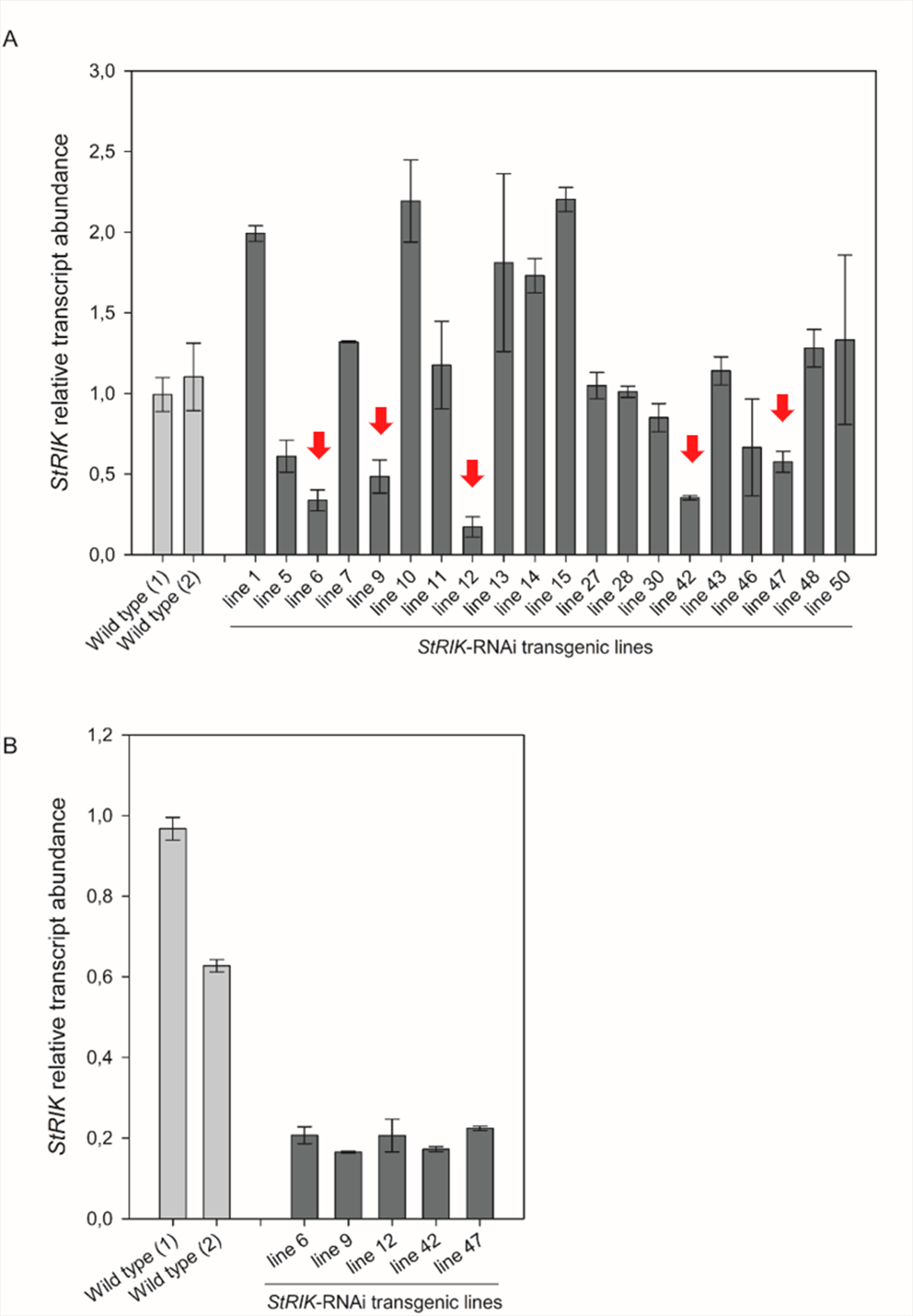

Supplement: Supplementary file 5 — Additional file 5: Fig. S5StRIK transcript accumulation measured by RT-qPCR in potato a) leaf and b) tuber periderm of StRIK-RNAi and Wild type lines from two consecutive plantings. Each column represents the mean and standard deviation of three technical replicates. Lines selected in leaves for further testing in tuber periderm are indicated with a red arrow. [file 12870_2021_3141_MOESM5_ESM.tif]
