# Supplementary material for: Silencing of StRIK in potato suggests a role in periderm related to RNA processing and stress
Source: BMC Plant Biol. 2021 Sep 7;21:409. doi: 10.1186/s12870-021-03141-z (PMC8424952; doi:10.1186/s12870-021-03141-z)
Supplement: Supplementary file 3 — Additional file 3: Fig. S3 Genomic StRIK sequence. Nucleotide sequence used for StRIK silencing is shown in red characters. The 12 exons of StRIK gene are shadowed in grey. The start initiation and the STOP codons are highlighted in green. [file 12870_2021_3141_MOESM3_ESM.docx]

**ATG**ACGGAGGATAATTGCCCTAGAGTTTCATCTTCTGAAACCGTAGACTCCAATTCTTCATCAACGAAGCAAAGGTGCATAAAAATTTCAGTTTTTAATCTTAGTTTTTTGTATGCAAACCACTTCAATTGAGATCTAAGTTTCCCTTCATATCATCGACTTAGGTCAAAATTGAAACTTTTCACGTATGATTAATTTTGTTCGCTATATCCTGAAAAAATATATTGTCTCTATAGTTTGGGATTGAGCCTAGTTGTTTCATACATTGATTGGTCCCCTCAACTTTTTTGCTGAGAAGCCCATTCCGGAACCCTATTAGCTCCGCTGGTATAATATGTTAGTAGAATACTACTTTGATTTCTTTCCAAAAAGTAGAAACGAACAGTCACGTAGTTTTAAGTAAGCTGCCATGATTAGATACGGGTTTGACCTAACTCAGTAATTAACTTTGGTCCAAACTTTTTGGCTGATGCTGGAGTTGGATTATTGTTTATTGAGTTCGAGGTTTCGCATGACTGTCTCCCACTATTTTAATCCTTTTGATGTATTATTGGTTCCGTGAGTTTGTCAGTGTCCTTTTTGCTCTGATGCTAAAATGAAAAAGTTCGGCAACTGAATTAGTACTTTGTTGAGATGAAATTGTTACTCTGTGGGAAGAAGCAGTATTGAGGTAATGGCAGTAGTAGTATTTTCACCGGGAAGTTACTGTTTTTTAGGCATTTAGAGAATCTGGGTTAACATAATAAATATTAGATGGATTGTTTGCATTATGCTGCAGAAGAGGAAAGAAAAATAAGAAACAACAACATACACAATTTGATTGATTGATTAATTAATATTAGAAAAATGATGAGAGACAGCTTTGTAGTCTAATCAGAAGAGTAAGCTCTTCTGGTGGTATAACCTTTCAACATCTTCCAGGAAACGGAGAAAGTGGGATCAGCCTGCGGAGACATTTGTTCCAGAAGGGGTAGCAGTATCTGGGATTTTTCCATTGGTAAATACTGGTTCTCTTGCTGGTATCACACTACCTGGCGTAATTCCAGTCTTGGGTGCTGCTTTTACGAATCCTCTTACCGCTATTGGTGCTACTACAGTGCAGCAGCTTCCCGTAATTATTGCCCAAAAATCAGTTCAAGTAAGATCATCAGAGAAGATGTTGCTTATTATTTTCTTGTTTGGAAGTATTCCCCTATTACATGAGGTTGTCACCTAGCTCTAGTAGTTATTATCCAAATTCATGATTTACATTCTCCCAATATTTGCTTTTCTTCCTTTTCTTTGTGGGTGGAAGAAATTATTTTTGTTGTCAGTAAGCGTTGCCCAATTATCTGCCTCCTTATATCATCATCAAAAAGAACAAGTTCTCTGGTTCACTTCCTGCAGTTTTCTTACTGCAAATTAACAGTAAATGTGTTTGTTACCCATCCTAGGAAATTATACTGAATCAGGCATGTTCTTTGTGTCATTTTTCCATTACCCTTCTACGAAATGCTGTTCAGAATCAGCTCTATTTTTTGTTTGGATCACTTCTGATTTTCCAATGAGCTATTGCAGCCGAAAATTCAGGATGAGTTGATAGCAAGAGAAATTGTCATTAATGATGCTGATCCGTCTGTTCGCTACAGGCTCACAAAGCGCCAAACACAGGAAGAGGTGAATATTGTTAATGGGTTTTGTAAGACTAAAGATGACTCGAGAAGCAAATGTGATTATCTTAAGGTTATGCTCATTATTTATTGATCAATTATCACTGTTTCTGCAGATTCAGAAGAGCACGGGTGCTGTGGTTATAACTAGGTAGCACACCATGAAGTCTTATTATATTGCTTATTGTTTAGCCTTTATTTACAGTGTGATACTTATTTAACGTGGCTATTGCAGGGGTAAATACAAGCCTCCAAGCGCACCATCCGATGGTGAAAAACCCCTATACCTTCATATTTCTGCAGGGGCACATGTAAGCAAGTTTAGTTACTTGCGTTTGGATGCACCATTTGGTTCAGGAAGATTGCTGATAAAGTTTTGTCCATCACTAAATTGCATAGATGAATCATTTTTCTGTGCATTTTTAATTAATCAATGCATTTGCCTTAGTGTCAACTTGTTTCTCTGAGTTTGGTTTCTGTGTTTTTTCCAACCATTGAAGTGGTTACCACCTATATTCTTCTGCTCGTCTTCTCTAGTTAAGAGTTCATTTACTGCTAAATTTTTTTCCCCGTTTTTATCCAATGTATACTACACATGACTGGAGAATCTGGAAATATCATTTCTCTTTAAAAGCAAAACCCAAATCATATTCCTATCTTCTGAACCTTTTCTACTTTTTTTAAAAAAATGTCTACACCTTTCATCTATCCAGCCAAAATTTAGTGTACATGTTTGGCTTGGCAAAGTTAGCGAGAGTTGAAAACATAAAAGTATAAGGTTAGAAATCCATTAATAACCTTTTGCTTCTTAGGAACAACAACAACAACATTACCCAGTAAAATCCCACAAAGTGGTGTCTGGGGATGCTATCTTAGGAACAAAATTAAAGAAAACAGTTGAGTGGAGAAATGTCTCATTAAGTTTCAGGCTATGTTATATTTTATAAAACACTTCCCATAGGGAAATTGTACTTATCTTGGGTCACACTATGAAGAAAAAATTGTTATTCATTAAAGTGGGACTTCTCTCTGTAACCTTTCAAGTTTCATTATTTTGACCTTTTACTTTTCTGGGTGAAGTGATTGCTATGGTGTATTTACATGTACTGTGGTTGACTCAGCTAGAAACAACACTTGAGAGGATCAGAGCAGTTGATCGTGCTGCTGCTGTAGTGGAGGAAATGCTTAAGCAAAGTCCTGTTAACAATGGATTAAAGGTAAGTAGTATCTACTGAGGTTTGACAGGACTTGAAGCCAGAAAATTACTGGAACCTCTGACTCTCTTATCATCAGGTTAATCATCTGCTGAGTACCTGTGTGTACCTGGGCTTTGAGGCTGATCCATCAGCAAATATTACTGTGCGCATTCGTGGACCAAATGTGAGCTCCCTTAATCTAACTTTGCTCTTTTCGCCTGGTTTATTGAGTTATTGTGGTATGTTGCCTATCAAGGGTGAATTTCCTTATAAAATTTTCGATTTATGAATTCTCGCATATTTAACGACATAAAAATAGGATCCCTTTTTTTGTGATTTACTATCCATTTGCCTTATCAATTTCTTTTCCCTATTTGTTGTGATTTGCTGGCAAATTTAGATACCGGGGAAGTTCATTCAGCTGTTACTTCTCTGTTATTGATCAGATATTGGCATCATTTATTCTCTTTATTGATCAGATTCTGGTCTGAGTAATTATATGATTGTTCCTTCCAGTAGTTTGGGTGTAGTCCATTCTCCACCCTGCCTGGCTAATTATCTTATTTATCTCTTTTGGCATTATGATGCTTGATGTGCTCCTTTAAACTTTATTCTTATTGATAGAAAGCCAAGAGATTCTAATGTTCTGAGCTCTCTCTGACATCTCCGTCCATTTGGAGAGATTTTCTTCTGTGCTGGTGGTTGGCTTTTTGTAAGTGCAGGATCAGTATATAAATCACATTATGAATGAAACTGGAGCAACTGTCTTGCTAAGAGGACGTGGTTCGGGATATTCAGATGAAGGAGAGGGAGAAGGTGCCTATGTGTTGATTAAGATGCAATTATTTTCCCATGCTCCATAGCATGATAAGAATGGTTATCAATAGTGTGCCACTGGCTAGATTTCTCAGCTATGAAAAAAATTTAGTTATTCCTCATAAATGTACTCTTTTAATGTGCAGATGTGCACCAACCTCTGCATTTACTCATATCAAGCAATAACAGTGCAAGTCTTGAGCGTGCGAAGCTTTTGGCAGAAAATCTTTTGGATACTATTTGTGCAGAGTGTGGTGCTTCTAGGTATGCATGTTGCTCTGAGTATATGAAATGTGATTGCAACCTGTTAGCTTTCGTTCAGAAGAGGTTTATGTGGGCTGTTATTTTGTTATGATTCTTTTTCTTTTATTGGGCTGAGTCCTATATCCTCATATGTGTGACTGTCGATCTGCAGAGTCTCTTCTTGTAAGGTTTATGGAGCTGTTCCGCCTCCACTGCAACCGTTAGCCAGCGTTCAGGTTTCTGGAAGTGAATCAGAAGTCAATAACATACCCACAGCCAATGTAGCTGCACAGATTTTGAGCTTCTCAACAGCAGCAGCAGTTCCCGTGACTGCAGCTGCAGGTGTGACTGGTGTTGTTTCTCAGGGTACAGTACCTCAATCTCTAGGCTCACTGAATCCTGTGCCATCTCAACCTCCCACCAGTTGCTATCCTCATCAATTAGTTGCAAGTAGAACAAGCTATATTGGTTATGATGGTATATATCCCCAAGCCACTGCTTTGCAGCAAGTTGCTTTGGCTCTTAGGCAATCTACTTCTCCAGTCACTACCACAGTTCCTCCAGCAACAACGGGACCAAGCATCACTTCACAGACAAGTACAGGTACTGAGAAGGACAAGCGCCCTGCACAGAAGCGTAAATTTCAAGAATTGCCGGCTGGTGGAAAAGGCCAATCAACTGTAAACCAGGTATGTGCTTAGTCTCCTTGACCTATACAAAGGTATCTCCAGGCAATTGCCCTTAACTGGCTTTTGATGAGAAAATTCTAATGGTCATCTTCGAGTCAAGTACATAAAGCTTCATGATTGACCAGAGATGGGGAATGAATATTGAAAGCATTTCATGTTATAATTTCTGGCATATGAACCTAAACTTGAGTATCCAGAAGTTCCTCCATTATCACTATCTGCTTGTTAAAGTATCCAATGGGGCACTGGCTGTGCAAAAAGTTTTGCTCCAAGCCTCCAAAATAAGCCCATCTCTTGGATGAGAACTAAAATGGCAGATGATATCATGTAAAAAAAAAGAAATAAGAAAAAAAGGGGGAACTATAGATGATATATTAGTACTGCCATTGCTACTGTTCCAACTCAATTTAGTGGAAGATTGAGGGGTACATGTATCACTAATCTGAATGGATCAAAAGCATCTCTAAGTACTTCTGTTTAGCCATTTATTGACCACCCACTATGTGGTATCTATCAGATCGACTGCTAGAGTGACAATATTACTGAAGAAAGATGTTGTTTAATGTGAGACAAGTTTCCCCTAGTGGTTGTGACTAGAGGTCACTGAAGTTGTATTGTTTCCCATTCCTCATTTGGAGGGCCTCCACCAAATCACGAAGTTCCTGTGATTTCTCCAAATTTATGCTCTGAAACAAAGCCTTCATAAACTGTGGTGCTATGTGGCATATCTATAGCCTGTGCATCTGCCTTGTTATATCTGAAGTTGTGTCCCTTAATTGCCTTTAATTGATTCTCACTGCTTGAGTGATCTTCTGCACAGGAATATGCTGTGTATATGAATTATGATAGATTCTTCACTTTACCTTCCACTATATAAATGGTATTAATAGAAGGATGAAGTACAGCTTGTGTAGCATGATGACTGGCAAAATTGGGATTTATCAGATTCTCTTATTTTTGGGTCTCTTTTAAACTTAGTTTACCATTCATCCTACATCTTAGTTTCTATTAAAAAAAGACTTTGTTGTTTCTCATTTTAAATTTTTATCACATTGCTGTTGTAAATGGAGCATTTGAAATTTCTCAAATTTCCTTCCCTTAAGATATTTTTCAGTTAAAAACATATATTTATTTCTGGTCTAACCTTTTCTACTTGATATTCCAGGTAATTTCTCTGACCTTGTATGAAGCTAACAGGCTGTGGAACTCTGTTGGACCTTTGTATGTTTCACAGGATTCTGGATACGGAGGAGGCATGGGGTTGCTACGTGCTATTTCAGTTTTATGGTGCCGATGCAAAGATACCATGCCCCTTCTGGTTTTGGCATTCTGTCAAATGTTAATATGCTAATATGTGCACTTTATTAATCAACTTTGCTTCAAGTACTAAAAGGGTAATGAGATCGAGATCACAATACATTCTGCCTGTGAGGCTTTCAAACTGTTTCAACTTTACTCTAGAAGAGAAAATGTTTTCTATTGGTATCTGCAATATATGCATTTTCTAAACATGATGATCTAAGCTCCTTGTAGCAAGTGATAGAAAAAAAAAGTCAAGCTGCAAAGTTGAAACAGTCCAATTTATTCATGTCAACCAATAATTCTTAGGATCATGCATTTATTTTAAACTTCCCTTGCCTGCTACTATACAAGTAAAATAATGTTAAAAGTATGCTCTCGAAAAACTCAAATTTTTATCTCCTTTTTTCCTGGAATTTCTAAGTTTTCTGGATGGACTCTCCCTCCCCCCTTTTGGTTGCCAGAATCCACTGCAGGGTACGGAGCTTCTAATGCTTCAGGAACGAATGTCAGACAAAGGTGATAGAGACAAAATTGGTACTCCGGCTCCCAGAAAGTTGGTTCAGCCCTTATCCAGTTCAATGCTGCCGCCGCCACCTCCTAGAATGATGCCTCCACTGCCACCGCCACCAAAGTTTCAATCATCCTCACAGAAAGTGCATGACAACAATGTGGTTAATAAAGCACCATGTAAAATTGTTCCAGGTATTTGCCATCACTCAAGCTTTTTCAGTCTCAAGATTTCAATAAACTCTTGCTTGGTTTGTCTATTAGTGATGACCTACCTATCTTCATTACCCCTTTTTTTCTTGCATTTCTTGTTACTAAGTGGAAGTGTGAAAATCCATTAGGTCCAAGTAACATAGTAGAACTTCTTTTACTTTTCATTTTTATCTTCCACTTCTTATTTTTGTATTATATCAGTAAGTATGAGATCAGCTTAAATGGGTTGAAGTTCACTGAAGATCTATATAAGAGACCCCAAGTAGCTTAGGATTGAGGCCTTGTAGTAGTTGTTATCAGACATAACTTGTCTATTCCATTTTTACAGTTGAGAAATATGAGAGTGTGTGTATTCAATTGTTTGATTTGATTTGTGCATCTCAGATACATTAGTCCAGCTAATGGAATATGGGGATGACGACGACGATGATAATGATGAAGCAATTGACGGACCTTTGAAATGTAGCTCAAGTGCGGTAGCAACTCCAAAGCCATTCTGGGCTGTTTAA
