# Supplementary material for: Silencing of StRIK in potato suggests a role in periderm related to RNA processing and stress
Source: BMC Plant Biol. 2021 Sep 7;21:409. doi: 10.1186/s12870-021-03141-z (PMC8424952; doi:10.1186/s12870-021-03141-z)
Supplement: Supplementary file 6 — Additional file 6: Table S1 BLASTN results against potato transcript database with the StRIK-RNAi construct as a query. [file 12870_2021_3141_MOESM6_ESM.docx]

| **Table S1.**  BLASTN results against potato transcript database with the *StRIK*-RNAi construct as a query. | | | | | |
| --- | --- | --- | --- | --- | --- |
| Accession | Description | Length (bp) | E value | Coverage (%) | Identity (%) |
| PGSC0003DMT400064729 | Protein RIK | 246 | 1E-137 | 100.00 | 100.00 |
| PGSC0003DMT400064730 | Protein RIK | 246 | 1E-137 | 100.00 | 100.00 |
| PGSC0003DMT400064731 | Protein RIK | 227 | 1E-126 | 92.28 | 100.00 |
| PGSC0003DMT400055483 | Conserved gene of unknown function | 18 | 0.23 | 7.32 | 100.00 |
| PGSC0003DMT400014640 | EMB1865 | 18 | 0.23 | 8.94 | 95.45 |
| PGSC0003DMT400014644 | EMB1865 | 18 | 0.23 | 8.94 | 95.45 |
| PGSC0003DMT400014643 | EMB1865 | 18 | 0.23 | 8.94 | 95.45 |
| PGSC0003DMT400030423 | Extensin | 17 | 0.92 | 6.91 | 100.00 |
| PGSC0003DMT400042918 | ATP binding protein | 17 | 0.92 | 6.91 | 100.00 |
| PGSC0003DMT400073845 | Dnajc14 protein | 17 | 0.92 | 6.91 | 100.00 |
